# Synthesis and Evaluation of Novel Benzofuran Derivatives as Selective SIRT2 Inhibitors

**DOI:** 10.3390/molecules22081348

**Published:** 2017-08-14

**Authors:** Yumei Zhou, Huaqing Cui, Xiaoming Yu, Tao Peng, Gang Wang, Xiaoxue Wen, Yunbo Sun, Shuchen Liu, Shouguo Zhang, Liming Hu, Lin Wang

**Affiliations:** 1 College of Life Science and Bioengineering, Beijing University of Technology, Beijing 100124, China; zhouym@imm.ac.cn; 2 Institute of Radiation Medicine, Academy of Military Medical Sciences, Beijing 100850, China; peng-tao@sohu.com (T.P.); wgang85@126.com (G.W.); crystalcat@vip.sina.com (X.W.); sunyunbo0919@126.com (Y.S.); liusc118@sohu.com (S.L.); 3 Institute of MateriaMedica, Chinese Academy of Medical Sciences & Peking Union Medical College, Beijing 100050, China; hcui@imm.ac.cn (H.C.); mingxyu@imm.ac.cn (X.Y.)

**Keywords:** benzofuran, sulfoxide, sulfone, selective SIRT2 inhibitor

## Abstract

A series of benzofuran derivatives were designed and synthesized, and their inhibitory activites were measured against the SIRT1–3. The enzymatic assay showed that all the compounds showed certain anti-SIRT2 activity and selective over SIRT1 and SIRT3 with IC_50_ (half maximal inhibitory concentration) values at the micromolar level. The preliminary structure–activity relationships were analyzed and the binding features of compound **7e** (IC_50_ 3.81 µM) was predicted using the CDOCKER program. The results of this research could provide informative guidance for further optimizing benzofuran derivatives as potent SIRT2 inhibitors.

## 1. Introduction

In humans, 18 histone deacetylases (HDACs) have been identified, which can be subdivided into four classes (HDAC Classes I–IV) primarily based on their homology to yeast HDACs [1,2]. Classes I, II and IV HDACs are referred to as “classical” HDACs and are dependent on Zn^2+^ for deacetylase activity. The only exception is the “unique” class III HDAC, or Sirtuins, as they require NAD^+^ (Nicotinamide adenine dinucleotide) instead of Zn^2+^ as a cofactor [3,4]. There are seven human Sirtuins (SIRT1–7) that present diversity in cellular localization and function [5]. SIRT1 is mainly a nuclear protein, while SIRT2 predominantly resides in the cytoplasm, SIRT3-5 are mitochondrial, but SIRT6 and SIRT7 are localized to the nucleus. They have been shown to have a major impact on cell cycle regulation [6], peripheral myelination [7], autophagy [8], immune and inflammatory response [9,10,11]. A dysregulation of SIRT2 activity was reported to play a critical role in the pathogenesis of cancer [12,13], neurodegenerative diseases [14], type II diabetes [15], and bacterial infections [10,11]. Therefore, SIRT2 inhibitors have been considered as candidate therapeutic agents of the diseases.

**Figure 1:**
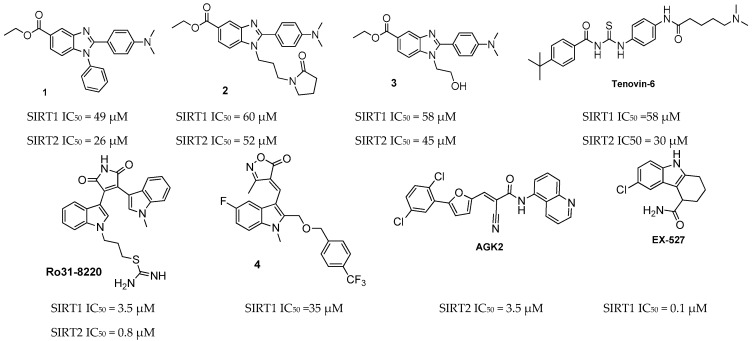
The chemical structures of some known sirtuin inhibitors.

Several classes of small molecule sirtuin inhibitors have been identified so far including nicotinamide derivatives, hydroxynaphthaldehyde derivatives, thiobarbiturate derivatives, tenovin derivatives, indole derivatives and benzimidazole derivatives, and most of the sirtuin inhibitors are targeting SIRT1 and SIRT2 (Figure 1) [16,17,18,19]. Among them, Tenovin-6 was identified as an inhibitor for SIRT1 and SIRT2 at a low micromolar level, it also inhibited growth in all tumor types in the study. AGK2 (2-cyano-3-[5-(2,5-dichlorophenyl)-2-furanyl]-*N*-5-quinolinyl-2-propenamide) possessed selective SIRT2 inhibitory activity. EX-527 showed remarkable SIRT1 inhibition potency with an IC_50_ (half maximal inhibitory concentration) value of 0.1 µM, structure modification of EX-527 has resulted in compound **4** with moderate SIRT1 inhibition potency and pharmacological properties, and compound Ro31-8220, a bis(indoly)maleinimides andinitially discovered as a kinase inhibitor, was found to inhibit SIRT1 and SIRT2 for IC_50_ value of 3.5 µM and 0.8 µM in later studies. Compounds **1**, **2** and **3** were reported to be moderately potent SIRT1 and SIRT2 inhibitors, and to exhibit antitumor activity on colon cancer cells and breast cancer cells. EX-527, compound **4**, and Ro31-8220 were based on the indole structure, compounds **1**, **2**, **3** bearing benzimidazolescaffold, and they shared some similarities. AGK2 was a furan scaffold, Benzofuran is isosteric with benzimidazole and indole, based on the structural features, we envisioned that the benzofuran scaffold may be a preferable sirtuin inhibitor. Therefore, we designed and synthesized a series of novel benzofuran derivatives, in which a methoxy or fluoro group was attached onto the 6-position, and a substituented benzyl sulfoxide or sulfone was incorporated into the 2-position. Here, we presented the synthesis and enzymatic inhibitory activities against SIRT1–3 of the benzofuran derivatives. The preliminary structure–activity relationships were investigated as well.

## 2. Result and Discussion

### 2.1. Chemistry

**Scheme 1:**
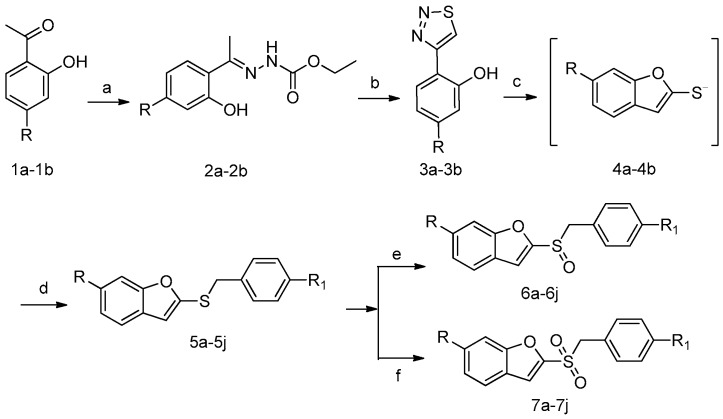
Synthesis of compounds **6a**–**6j** and **7a**–**7j.** Reagents and conditions: A ethoxycarbonylhydrazine, concentrated HCl, ethanol, 80 °C; b SOCl_2_, CHCl_3_ and DMF (*N*,*N*-Dimethylformamide); c K_2_CO_3_, DMF, 100 °C; d 4-substituted benzyl bromide, K_2_CO_3_, DMF, 30 °C; e m-CPBA (3-Chloroperbenzoic acid), CH_2_Cl_2_, 0 °C; **f** m-CPBA, CH_2_Cl_2_, r.t.

We adopted and modified the protocol to synthesize benzofuran derivatives from previously published literature [20,21,22], the synthesis of target compounds **6a**–**6j** and **7a**–**7j** was depicted in Scheme 1, and their chemical structures were shown in Table 1.

The mixture of compounds **1a**–**1b**, ethoxycarbonylhydrazine and concentrated HCl in ethanol were heated to give rise to the intermediates **2a**–**2b**. 1,2,3-thiadiazoles **3a**–**3b** were obtained from **2a**–**2b** with subsequent treatment with thionyl chloride. In the presence of potassium carbonate, the key intermediate benzofuran-2-thiols **4a**–**4b** were prepared by the intramolecular cyclization reaction, which were further transformed into compounds **5a**–**5j** by the alkylation with 4-substituted benzyl bromide, the oxidation of **5a**–**5j** with 1.1 equivalents *m*-chloroperoxybenzoic acid (m-CPBA) at 0 °C to generate the corresponding sulfoxides **6a**–**6j** or with an excess of m-CPBA at r.t. to form the sulfone target compounds **7a**–**7j** in good yield (over 90%).

During the course of compounds **3a**–**3b** synthesis, we initially used the reaction conditions according to the reference [22], and the mixture of compounds **2a**–**2b** and thionyl chloride was heated in chloroform for 1–2 h at 60 °C to afford compounds **3a**–**3b**. We found that compounds **3a**–**3b**, particularly **3a,** had poor yield about 30%. Thus, we tried to optimize the reaction conditions as shown in Scheme 1. In this condition, the solvent was the mixture of chloroform and DMF (5:1), the reaction started and finished by releasing heat with regulation of the rate of dropping thionyl chloride to giving rise to the intermediates **3a**–**3b** over 90% yield. Then, **3a**–**3b** reacted with potassium carbonate and 4-substituted benzyl bromide, forming the key intermediates **5a**–**5j** and quantitative reaction in one-pot reaction. In fact, compared with the method of reference, our approach was more efficient and concise.

### 2.2. Sirtuin-Inhibitory Activity Evaluation

All target compounds were screened against human recombinant SIRT1-3 using AMC (Coumarin)-tagged deacetylation substrates [23]. Tenovin-6 is one of the few sirtuin inhibitors that displayed antitumor activities in a mouse xenograft model. AGK2 is one of the most selective SIRT2 inhibitors to date, and reduced α-synuclein-induced cytotoxicity in a Parkinson’s disease model. Thus, they were used as the standard control for SIRT1–3 assays. IC_50_ values were determined for all of the compounds.

**Figure 2:**
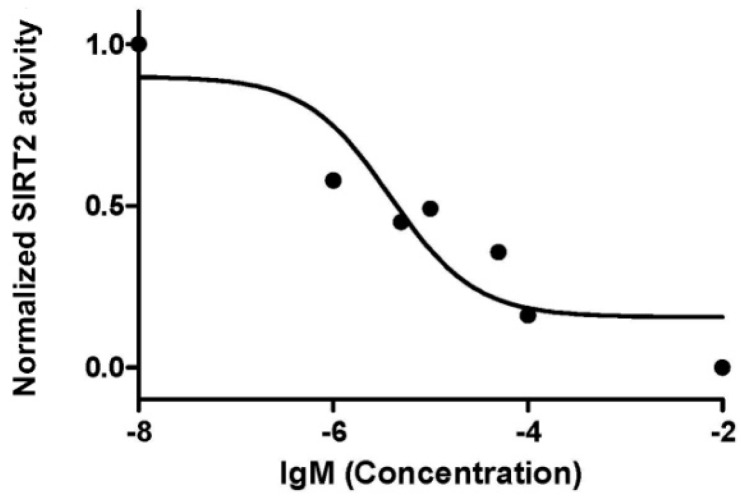
SIRT2 inhibition dose response curves for the most potent compound **7e**.

To explore the SAR (Structure activity relationships) of the substituted benzofuran core, the electron donating groups (methoxy-) and electron withdrawing groups (fluoro-) were chosen as the substituent attached on the 6-position of benzofuran moiety. A substituted benzyl sulfoxide or sulfone was incorporated into the 2-position. Substitution of benzenering was based on previous benzimidazole work [19], and those that showed high sirtuin inhibitory activities were substituted at the para position of benzene ring. Therefore, methoxyl, cyano, bromo, fluoro and methoxycarbonyl were added at the 4-position. In vitro screening on all of the target compounds **6a**–**6j** and **7a**–**7j** exhibited selective SIRT2 inhibitory activities with IC_50_ varied from 3.81 µM to 95.21 µM compared to SIRT1 and SIRT3 with IC_50_ more than 100 µM. Compound **7e** (IC_50_ 3.81 µM) was the most potent SIRT2 inhibitor, being more potent than Tenovin-6 (IC_50_ 15.32 µM), and SIRT2 activity dose-response profiles for compound **7e** are shown in Figure 2. Compounds **7a**–**7j** with benzyl sulfone scaffold possessed the better SIRT2 inhibitory activities in comparison with the corresponding benzyl sulfoxide compounds **6a**–**6j**, which are chiral sulfones. Chirality has revealed to be crucial for other SIRT inhibitors as EX-527, in view of the structural differences and the general activity trend of such compounds. We speculate that the SIRT-inhibitory activity of them is related to chirality, and this deserves to be studied further. Among compounds **7a**–**7j**, most compounds showed good potency, and they had IC_50_ values at single digit micromolar level (IC_50_ 3.81–8.85 µM). The inhibitory activity of the 4-substituted derivatives on benzene rings increased in the order of CH_3_OCO- > CH_3_O- > CN- > Br- > F-. It was noted that 4-broro substituted compounds (**7c**, **7h**) had somewhat lower activity (IC_50_ 17.76, 20.14 µM), and 4-fluoro substituted compounds (**7d**, **7i**) displayed the weakest inhibition on SIRT2 (IC_50_ 43.93, 51.42 µM) in this series. Collectively, it suggested that the 4-halogen substituent of the benzene ring might be unfavorable for potency. In addition, when the methoxyl group is substituted for the benzofuran moiety as R-group, and compounds **7a**–**7e** possessed the better inhibitory activity than that of compounds **7f**–**7j** with a fluoro group. This result gave an indication that the inhibitory effect was enhanced when an electron donating groups was added to the benzofuran core. Compounds **6a**–**6j** have a similar structure–activity relationship to compounds **7a**–**7j**.

As mentioned above (Figure 1), compounds **1**, **2** and **3** bearing the benzimdazole scaffold were generally unselective with regards to SIRT1 and SIRT2 with inhibition potency. The sirtuin inhibitors of compound **4**, Ro31–8220, and EX-527 with indole moiety were shown to have certain selectivity for SIRT1 and SIRT2. We found that most of the *N*-containing heterocycles target SIRT1 and SIRT2, while benzofurans and furan (AGK2) preferentially target only SIRT2.

**Figure 3:**
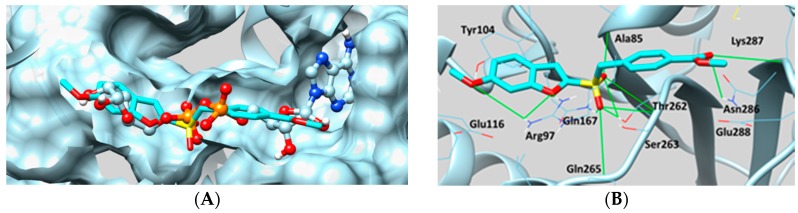
CDOCKER-modeled binding mode of compound **7e** (carbon atoms colored aqua, oxygen atom colored red, sulfur atom colored yellow) with SIRT2 (3ZGV in PDB). (**A**) the binding pose of compound **7e** within the binding site of the ADP-ribose (carbon atoms colored powderblue, nitrogen atom colored blue, phosphorus atom colored orange, oxygen atom colored red); (**B**) the interactions of compound **7e** with key amino acids within the binding stie. H-Bonding interactions were presented with green lines.

To explore potential binding modes of these novel benzofuran derivatives, molecular docking was performed using CDOCER protocol integrated in Accelrys Discovery Studio Client 2016 (Accelrys Software Inc., San Diego, CA, USA). The coordinates of the X-ray co-crystal structure of ADP-ribose (ADPr) with SIRT2 (PDB entry code: 3ZGV, X-ray resolution = 2.30 Å), reported by Moniot et al. in 2013 [24], was employed for docking the representative compound **7e** (the most active compound). The binding modes of the benzofuran derivatives were somewhat similar and exemplified by compound **7e**, as shown in Figure 3, and compound **7e** could nicely situate in the binding pocket and took a very similar binding pose with the ADPr.

The docking analysis reveals that key interactions of compound **7e** with the active site of SIRT2 are the hydrogen bonds as well as the π–π stacking interactions. The benzofuran moieties involved in potential π-π stacking with Tyr104. The oxygen atom of benzofuran moiety interacted with Gln167 and Arg97 via a strong hydrogen bond as we intended. The co-crystal structures of known SIRT2 inhibitors have demonstrated that the hydrogen bonds between Arg97 and Gln167 are crucial to inhibit the function of SIRT2 [25,26,27]. Similarly, our docking results have also shown hydrogen bond interactions with Gln167 and Arg97. Therefore, the benzofuran moiety played a critical role in the potency. It was noticed that the sulfone oxygen on the 2-position of the benzofuran scaffold formed strong hydrogen bonds with Ala85, Thr262, Ser263. Other hydrogen bonds that could be observed include Gln167 and Gln265, and these suggested that the oxygen atom on the 2-position was benefical for the binding. On the other hand, the binding pattern suggests that the configuration of the 2-position oxygen atom determines the intensity of the hydrogen bond. In fact, the corresponding compound **6e** bearing a sulfoxide group showed a poorer inhibition effect (IC_50_ 15.68 µM) than that of compound **7e**. Similarly, all of the other compounds with a sulfoxide group had lower SIRT2 inhibitory activities, in comparison with the corresponding sulfone compounds in Table 1. The results prove our speculation that the chirality of sulfoxide affects its inhibitory activity. In addition, the 6-methoxyl oxygen on the benzofuran core also formed a hydrogen bond with Arg97, and the 4-carboxylate oxygen of benzene ring formed hydrogen bonds with Lys287 and Asn286. These may add more favorable binding affinity.

## 3. Experimental Section

### 3.1. Chemistry

Commercial grade reagents and solvents were used without further purification. Melting points were measured on a Fisher Scientific (Waltham, MA, USA) micro melting point apparatus and are uncorrected. ^1^H-NMR spectra was recorded on a Varian Mercury 400 MHz (Palo Alto, CA, USA) spectrometer with tetramethylsilane (TMS) as the internal standard for ^1^H. Chemical shifts are reported in δ (ppm) units. ^13^C-NMR spectra were recorded using a Bruker 600 MHz NMR (Billerica, MA, USA) spectrometer. High-resolution mass spectra (HRMS) were obtained on a 9.4T Q-q-FTMSApex Qespectrometer (Karlsruhe, Germany). All reactions were magnetically stirred and monitored by thin-layer chromatography (TLC) on pre-coated silica gel G plates (Qingdao Marine Chemical Factory, Qingdao, China) at 254 nm under a UV lamp. Column chromatography separations were performed with silica gel (200–400 mesh).

### 3.2. Synthesis of Compounds ***6a**–**6j***

*6-Methoxy-2-((4-methoxybenzyl)sulfinyl)*benzofuran (**6a**). To a stirred solution of **5a** (400 mg, 1.33 mmol) in DCM (dichloromethane) (15.0 mL) and cooled in an ice bath, m-CPBA (296 mg, 1.47 mmol) was added slowly at 0 °C. The reaction mixture was stirred at 0 °C for 30 min. The reaction mixture was quenched with aqueous saturated NaHCO_3_ (10 mL). The aqueous layer was then extracted with DCM (3 × 10 mL) and the combined organic layer washed with brine (20 mL), dried (Na_2_SO_4_) and concentrated in vacuo. The crude product was purified by column. Chromatography on silica gel eluting with petroleum ether/ethyl acetate/dichloromethane(*v:v* = 4:1:0.6) to yield the product (399 mg, 95%) as white flocky solid; m.p. 129–130 °C; ^1^H-NMR (400 MHz, CDCl_3_) δ (ppm): 7.45 (d, *J* = 8.8 Hz, 1H), 7.10 (s, 1H), 7.03 (t, *J* = 8.4 Hz, 3H), 6.94 (dd, *J*_1_ = 8.4 Hz, *J*_2_ = 1.9 Hz, 1H), 6.76 (d, *J* = 8.42 Hz), 4.53 (d, *J* = 12.4 Hz, 1H), 4.40 (d, *J* = 12.4 Hz, 1H), 3.90 (s, 3H), 3.75 (s, 3H) (Supplementary Figure S1). ^13^C NMR (151 MHz, CDCl_3_) δ (ppm): 159.93, 159.71, 157.73, 151.73, 131.32, 122.53, 120.96, 119.70, 114.20, 113.57, 95.92, 58.68, 55.75, 55.21 (Supplementary Figure S2). HRMS (ESI (electrospray ionization)) Calcd. for C_17_H_16_NaO_4_S [M + Na]^+^: 339.0667; Found: 339.0665.

*6-Methoxy-2-((4-cyanobenzyl)sulfinyl)benzofuran* (**6b**). Following the preparation protocol of compound **6a**, starting from compound **5b** (280 mg, 0.95 mmol), the title compound **6b** was obtained as white crystals (268 mg, 91%); m.p. 137–138 °C; ^1^H-NMR (400 MHz, CDCl_3_) δ (ppm): 7.55 (d, *J* = 8.0 Hz, 2H), 7.46 (d, *J* = 8.8 Hz, 1H), 7.26–7.22 (m, 2H), 7.07 (s, 1H), 7.02 (s, 1H), 6.98–6.94 (m, 1H), 4.53 (d, *J* = 12.8 Hz, 1H), 4.47 (d, *J* = 12.8 Hz, 1H), 3.90 (s, 3H) (Supplementary Figure S3). ^13^C-NMR (151 MHz, CDCl_3_) δ (ppm): 160.16, 157.83, 150.92, 134.54, 132.34, 130.85, 122.68, 119.43, 118.27, 113.82, 113.74, 112.41, 95.89, 58.79, 55.78 (Supplementary Figure S4). HRMS (ESI) Calcd. for C_17_H_14_NO_3_S [M + H]^+^: 312.0694; Found: 312.0687.

*6-Methoxy-2-((4-bromobenzyl)sulfinyl)benzofuran* (**6c**). Following the preparation protocol of compound **6a**, starting from compound **5c** (400 mg, 1.33 mmol), the title compound **6c** was obtained as white crystals (430 mg, 94%); m.p. 146–147 °C; ^1^H-NMR (400 MHz, CDCl_3_) δ (ppm): 7.46 (d, *J* = 8.8 Hz, 1H), 7.38 (d, *J* = 8.0 Hz, 2H), 7.08 (s, 1H), 7.04–6.97 (m, 3H), 6.94 (dd, *J*_1_ = 8.4 Hz, *J*_2_ = 1.6 Hz, 1H), 4.49 (d, *J* = 12.4 Hz, 1H), 4.39 (d, *J* = 12.8 Hz, 1H), 3.90 (s, 3H) (Supplementary Figure S5). ^13^C-NMR (151 MHz, CDCl_3_) δ: 160.06, 157.78, 151.26, 131.91, 131.70, 128.15, 122.79, 122.64, 119.56, 113.74, 113.71, 95.90, 58.55, 55.77. HRMS (ESI) Calcd. for C_16_H_14_BrO_3_S [M + H]^+^: 364.9847, Found: 364.9840.

*6-Methoxy-2-((4-fluorobenzyl)sulfinyl)benzofuran* (**6d**). Following the preparation protocol of compound **6a**, starting from compound **5d** (420 mg, 1.46 mmol), the title compound **6d** was obtained as white crystals (408 mg, 92%); m.p. 104.5–105.5 °C; ^1^H-NMR (400 MHz, CDCl_3_) δ (ppm): 7.45 (d, *J* = 8.8 Hz, 1H), 7.13–7.07 (m, 3H), 7.01 (s, 1H), 6.97–6.90 (m, 3H), 4.51 (d, *J* = 12.8 Hz, 1H), 4.41 (d, *J* = 12.8 Hz, 1H), 3.90 (s, 3H) (Supplementary Figure S6). ^13^C-NMR (151 MHz, CDCl_3_) δ: 162.78 (d, *J* = 246.8 Hz), 160.01, 157.76, 151.39, 131.8 (d, *J* = 8.3 Hz), 124.98 (d, *J* = 3.0 Hz), 122.58, 119.58, 115.77 (d, *J* = 21.6 Hz), 113.66, 113.62, 95.89, 58.37, 55.75 (Supplementary Figure S7). HRMS (ESI) Calcd. for C_16_H_14_FO_3_S [M + H]^+^: 305.0647; Found: 305.0638.

*6-Methoxy-2-((4-methoxycarbonylbenzyl)sulfinyl)benzofuran* (**6e**). Following the preparation protocol of compound **6a**, starting from compound **5e** (328 mg, 1.00 mmol), the title compound **6e** was obtained as white crystals (319 mg, 93%); m.p. 157–158 °C; ^1^H-NMR (400 MHz, CDCl_3_) δ (ppm): 7.91 (d, *J* = 8.0 Hz, 2H), 7.44 (d, *J* = 8.8 Hz, 1H), 7.20 (d, *J* =8.0 Hz, 2H), 7.09 (s, 1H), 7.00 (s, 1H), 6.97–6.91 (m, 1H), 4.59 (d, *J* = 12.4 Hz, 1H), 4.49 (d, *J* = 12.8 Hz, 1H), 3.90 (s, 3H), 3.88 (s, 3H) (Supplementary Figure S8). ^13^C-NMR (151 MHz, CDCl_3_) δ (ppm): 166.51, 160.09, 157.80, 151.18, 134.19, 130.17, 130.13, 129.91, 122.65, 119.51, 113.77, 113.75, 95.90, 59.02, 55.77, 52.18 (Supplementary Figure S9). HRMS (ESI) Calcd. for C_18_H_17_O_5_S [M + H]^+^: 345.0796, Found: 345.0791.

*6-Fluoro-2-((4-methoxybenzyl)sulfinyl)benzofuran* (**6f**). Following the preparation protocol of compound **6a**, starting from compound **5f** (350 mg, 1.22 mmol), the title compound **6f** was obtained as white crystals (330 mg, 89%); m.p. 140–141 °C; ^1^H-NMR (400 MHz, CDCl_3_) δ (ppm): 7.53 (dd, *J*_1_ = 8.4 Hz, *J*_2_ = 5.2 Hz, 1H), 7.32 (dd, *J*_1_ = 8.8 Hz, *J*_2_ = 1.6 Hz, 1H), 7.12–7.01 (m, 4H), 6.80–6.74 (m, 2H), 4.50 (d, *J* = 12.8 Hz, 1H), 4.38 (d, *J* = 12.8 Hz, 1H), 3.76 (s, 3H) (Supplementary Figure S10). ^13^C-NMR (151 MHz, CDCl_3_) δ: 162.08 (d, *J* = 245.6 Hz), 159.81, 156.40 (d, *J* = 13.5 Hz), 154.24, 131.32, 122.89 (d, *J* = 10.2 Hz), 122.83, 120.57, 114.22, 112.74, 112.73 (d, *J* = 24.3 Hz), 99.72 (d, *J* = 26.7 Hz), 58.90, 55.23 (Supplementary Figure S11). HRMS (ESI) Calcd. for C_16_H_13_FNaO_3_S [M + Na]^+^: 327.0467, Found: 327.0463.

*6-Fluoro-2-((4-cyanobenzyl)sulfinyl)benzofuran* (**6g**). Following the preparation protocol of compound **6a**, starting from compound **5g** (240 mg, 0.85 mmol), the title compound **6g** was obtained as white crystals (268 mg, 95%); m.p. 105.5–107 °C;^1^H-NMR (400 MHz, CDCl_3_) δ (ppm): 7.59–7.53 (m, 3H), 7.31 (dd, *J*_1_ = 8.4 Hz, *J*_2_ = 1.2 Hz, 1H), 7.28–7.22 (m, 2H), 7.13–7.04 (m, 2H), 4.52 (d, *J* = 12.8 Hz, 1H), 4.45 (d, *J* = 12.8 Hz, 1H) (Supplementary Figure S12). ^13^C-NMR (151 MHz, CDCl_3_) δ (ppm): 162.19 (d, *J* = 246.3 Hz), 156.46 (d, *J* = 13.4 Hz), 153.47, 134.14, 132.32, 130.84, 123.07 (d, *J* = 10.2 Hz), 122.59, 118.19, 113.06 (d, *J* = 24.3 Hz), 112.91, 112.54, 99.71 (d, *J* = 26.7 Hz), 58.83 (Supplementary Figure S13). HRMS (ESI) Calcd. for C_16_H_11_FNO_2_S [M + H]^+^: 300.0494, Found: 300.0488.

*6-Fluoro-2-((4-bromobenzyl)sulfinyl)benzofuran* (**6h**). Following the preparation protocol of compound **6a**, starting from compound **5h** (490 mg, 1.45 mmol), the title compound **6h** was obtained as white crystals (462 mg, 90%); m.p. 137–138.5 °C; ^1^H-NMR (400 MHz, CDCl_3_) δ (ppm): 7.55 (dd, *J* = 8.8 Hz, 5.6 Hz, 1H), 7.41–7.36 (m, 2H), 7.31 (dd, *J*_1_ = 8.8 Hz, *J*_2_ = 2.0 Hz, 1H), 7.13–7.06 (m, 2H), 6.99 (d, *J* = 8.4 Hz, 2H), 4.47 (d, *J* = 12.8 Hz, 1H), 4.37 (d, *J* = 12.8 Hz, 1H) (Supplementary Figure S14). ^13^C-NMR (151 MHz, CDCl_3_) δ: 162.17 (d, *J* = 245.9 Hz), 156.45 (d, *J* = 13.4 Hz), 153.80, 131.95, 131.69, 127.78, 123.07, 122.98 (d, *J* = 5.7 Hz), 122.72, 112.92 (d, *J* = 24.2 Hz), 112.91, 99.74 (d, *J* = 26.9 Hz), 58.70 (Supplementary Figure S15). HRMS (ESI) Calcd. for C_15_H_10_BrFNaO_2_S [M + Na]^+^: 374.9466; Found: 374.9457.

*6-Fluoro-2-((4-fluorobenzyl)sulfinyl)benzofuran* (**6i**). Following the preparation protocol of compound **6a**, starting from compound **5i** (480 mg, 1.74 mmol), the title compound **6i** was obtained as white crystals (489 mg, 96%); m.p. 107.5–109 °C; ^1^H-NMR (400 MHz, CDCl_3_) δ (ppm): 7.54 (dd, *J*_1_ = 8.4 Hz, *J*_2_ = 5.2 Hz, 1H), 7.31 (d, *J* = 8.4 Hz, 1H), 7.13–7.04 (m, 4H), 6.95 (d, *J* = 8.4 Hz, 2H), 4.49 (d, *J* = 12.8 Hz, 1H), 4.40 (d, *J* = 12.8 Hz, 1H) (Supplementary Figure S16). ^13^C-NMR (151 MHz, CDCl_3_) δ(ppm): 162.87 (d, *J* = 246.9 Hz), 162.13 (d, *J* = 245.8 Hz), 156.44 (d, *J* = 13.5 Hz), 153.95, 131.84 (d, *J* = 8.3 Hz), 124.63 (d, *J* = 2.6 Hz), 122.96 (d, *J* = 10.2 Hz), 122.74, 115.81 (d, *J* = 21.6 Hz), 112.94, 112.78, 99.72 (d, *J* = 26.7 Hz), 58.55 (Supplementary Figure S17). HRMS (ESI) Calcd. for C_15_H_11_F_2_O_2_S [M + H]^+^: 293.0447; Found: 293.0439.

*6-Fluoro-2-((4-methoxycarbonylbenzyl)sulfinyl)benzofuran* (**6j**). Following the preparation protocol of compound **6a**, starting from compound **5j** (310 mg, 0.95 mmol), the title compound **6j** was obtained as white crystals (308 mg, 95%); m.p. 137–138 °C; ^1^H-NMR (400 MHz, CDCl_3_) δ (ppm): 7.92 (d, *J* = 8.0 Hz, 2H), 7.53 (dd, *J*_1_ = 8.8 Hz, *J*_2_ = 5.2 Hz, 1H), 7.31 (dd, *J*_1_ = 8.8 Hz, *J*_2_ = 1.2 Hz, 1H), 7.22–7.17 (m, 2H), 7.09 (td, *J*_1_ = 9.0 Hz, *J*_2_ = 2.2 Hz, 1H), 7.04 (s, 1H), 4.57 (d, *J* = 12.6 Hz, 1H), 4.47 (d, *J* = 12.4 Hz, 1H), 3.89 (s, 3 H) (Supplementary Figure S18). ^13^C-NMR (151 MHz, CDCl3) δ: 166.44, 162.17 (d, *J* = 246.0 Hz), 156.44 (d, *J* = 13.5 Hz), 153.68, 133.78, 130.28, 130.11, 129.90, 123.02 (d, *J* = 10.2 Hz), 122.65, 112.91 (d, *J* = 24.2 Hz), 112.90, 99.72 (d, *J* = 26.9 Hz), 59.12, 52.18 (Supplementary Figure S19). HRMS (ESI) Calcd. for C_17_H_14_FO_4_S [M + H]^+^: 333.0596; Found: 333.0587.

### 3.3. Synthesis of Compounds ***7a**–**7j***

*6-Methoxy-2-((4-methoxybenzyl)sulfonyl)benzofuran* (**7a**). To a stirred solution of compound **5a** (400 mg, 1.33 mmol) in DCM (20.0 mL) and *m*-CPBA (675 mg, 3.34 mmol) was added slowly at r.t. The mixture was stirred at r.t for 30 min. The reaction mixture was quenched with aqueous saturated NaHCO_3_ (10 mL). The aqueous layer was then extracted with CH_2_Cl_2_ (3 × 10 mL) and the combined organic layer washed with brine (20 mL), dried (Na_2_SO_4_) and concentrated in vacuo. The crude was purified by silica gel column chromatography (ether/ethyl acetate 2:1) to afford the title compound **7a** (413 mg, 93%). white flocky solid; m.p. 114–115 °C; ^1^H-NMR (400 MHz, CDCl_3_) δ (ppm): 7.49 (d, *J* = 8.8 Hz, 1H), 7.20 (s, 1H), 7.10 (d, *J* = 8.4 Hz, 2H), 7.05 (s, 1H), 6.97 (dd, *J*_1_ = 8.8 Hz, *J*_2_ = 2.0 Hz, 1H), 6.79 (d, *J* = 8.8 Hz, 2H), 4.45 (s, 2H), 3.89 (s, 3H), 3.77 (s, 3H) (Supplementary Figure S20). ^13^C-NMR (151 MHz, CDCl_3_) δ 160.83, 160.17, 157.72, 147.54, 131.91, 123.39, 119.10, 118.94, 115.79, 114.54, 114.24, 95.79, 60.91, 55.77, 55.24 (Supplementary Figure S21). HRMS (ESI) Calcd. for C_17_H_16_NaO_5_S [M + Na]^+^: 355.0616; Found: 355.0613.

*6-Methoxy-2-((4-cyanobenzyl)sulfonyl)benzofuran* (**7b**). Following the preparation protocol of compound **7a**, starting from compound **5b** (650 mg, 2.20 mmol), the title compound **7b** was obtained as white solid (668 mg, 93%); m.p. 160–161 °C; ^1^H-NMR (400 MHz, CDCl_3_) δ (ppm): 7.59(d, *J* = 8.0 Hz, 2H), 7.52 (d, *J* = 8.4 Hz, 1H), 7.33 (d, *J* = 7.6 Hz, 2H), 7.27–7.23 (m, 1H), 7.04–6.97 (m, 2H), 4.55 (s, 2H), 3.90 (s, 3H) (Supplementary Figure S22). ^13^C-NMR (151 MHz, CDCl_3_) δ (ppm): 161.22, 157.83, 146.82, 132.56, 132.45, 131.44, 123.59, 118.62, 118.08, 116.37, 114.96, 113.15, 95.7361.18, 55.82 (Supplementary Figure S23). HR-MS (ESI) Calcd. for C_17_H_13_NNaO_4_S [M + Na]^+^: 350.0463, Found: 350.0456.

*6-Methoxy-2-((4-bromobenzyl)sulfonyl)benzofuran* (**7c**). Following the preparation protocol of compound **7a**, starting from compound **5c** (500 mg, 1.43 mmol), the title compound **7c** was obtained as white solid (530 mg, 97%); m.p. 133–134 °C;^1^H-NMR (400 MHz, CDCl_3_) δ (ppm): 7.51 (d, *J* = 8.8 Hz, 1H), 7.41 (d, *J* = 8.4 Hz, 2H), 7.23 (s, 1H), 7.08–7.03 (m, 3H), 6.98 (dd, *J*_1_ = 8.8 Hz, *J*_2_ = 2.0 Hz, 1H), 4.46 (s, 2H), 3.89 (s, 3H) (Supplementary Figure S24). ^13^C-NMR (151 MHz, CDCl3) δ (ppm): 161.02, 157.78, 147.08, 132.24, 132.01, 126.36, 123.56, 123.51, 118.77, 116.13, 114.76, 95.76, 60.88, 55.79; HR-MS (ESI) Calcd. for C_16_H_13_BrNaO_4_S [M + Na]^+^: 402.9615, Found:402.9610.

*6-Methoxy-2-((4-fluorobenzyl)sulfonyl)benzofuran* (**7d**). Following the preparation protocol of compound **7a**, starting from compound **5d** (580 mg, 2.01 mmol), the title compound **7d** was obtained as white solid (601 mg, 93%); m.p. 99–100 °C;^1^H-NMR (400 MHz, CDCl_3_) δ (ppm): 7.50 (d, *J* = 8.4 Hz, 1H), 7.22 (s, 1H), 7.20–7.14 (m, 2H), 7.04 (s, 1H), 7.01–6.94 (m, 3H), 4.48 (s, 2H), 3.89 (s, 3H) (Supplementary Figure S25). ^13^C-NMR (151 MHz, CDCl_3_) δ (ppm): 163.19(d, *J* = 247.5 Hz), 160.98, 157.76, 147.19, 132.48 (d, *J* = 8.4 Hz), 123.46, 123.19 (d, *J* = 3.0 Hz), 118.81, 116.02, 115.89 (d, *J* = 21.6 Hz), 114.70, 95.77, 60.70, 55.79 (Supplementary Figure S26). HR-MS (ESI) Calcd. for C_16_H_13_FNaO_4_S [M + Na]^+^: 343.0416, Found: 343.0410.

*6-Methoxy-2-((4-methoxycarbonylbenzyl)sulfonyl)benzofuran* (**7e**). Following the preparation protocol of compound **7a**, starting from compound **5e** (292 mg, 0.89 mmol), the title compound **7e** was obtained as light white solid (293 mg, 92%); m.p. 175–176 °C; ^1^H-NMR (400 MHz, CDCl_3_) δ (ppm): 7.94 (d, *J* = 8.4 Hz, 2H), 7.49(d, *J* = 8.8 Hz, 1H), 7.29–7.25 (m, 2H), 7.19 (d, *J* = 0.8 Hz, 1H), 7.04 (d, *J* = 1.6 Hz, 1H), 6.98 (dd, *J*_1_ = 8.8 Hz, *J*_2_ =2.0 Hz, 1H), 4.56 (s, 2H), 3.90 (s, 3H), 3.89 (s, 3H) (Supplementary Figure S27). ^13^C-NMR (151 MHz, CDCl_3_) δ (ppm): 166.41, 161.05, 157.77, 147.00, 132.25, 130.73, 129.92, 123.51, 118.73, 116.17, 114.77, 114.69, 95.76, 61.26, 55.79, 52.24 (Supplementary Figure S28). HR-MS (ESI) Calcd. for C_18_H_17_O_6_S [M + H]^+^: 361.0745, Found: 361.0737.

*6-Fluoro-2-((4-methoxybenzyl)sulfonyl)benzofuran* (**7f**). Following the preparation protocol of compound **7a**, starting from compound **5f** (400 mg, 1.39 mmol), the title compound **7f** was obtained as light white solid (410 mg, 92%); m.p. 119–120.5 °C; ^1^H-NMR (400 MHz, CDCl_3_) δ (ppm): 7.60 (dd, *J*_1_ = 8.8 Hz, *J*_2_ = 5.6 Hz, 1H), 7.30 (dd, *J*_1_ = 8.0 Hz, *J*_2_ = 2.0 Hz, 1H), 7.26 (d, *J* = 1.2 Hz, 1H), 7.16–7.08 (m, 3H), 6.80 (d, *J* = 8.8 Hz, 2H), 4.47 (s, 2H), 3.77 (s, 3H) (Supplementary Figure S29). ^13^C-NMR (151 MHz, CDCl_3_) δ (ppm): 162.85 (d, *J* = 247.5 Hz), 160.27, 156.31 (d, *J* = 13.8 Hz), 149.69, 131.90, 124.00 (d, *J* = 10.4 Hz), 122.07, 118.71, 115.27, 114.30, 113.57 (d, *J* = 24.5 Hz), 100.02 (d, *J* = 26.9 Hz), 60.88, 55.26 (Supplementary Figure S30). HR-MS (ESI) Calcd. for C_16_H_13_FNaO_4_S [M + Na]^+^: 343.0416, Found: 343.0409.

*6-Fluoro-2-((4-cyanobenzyl)sulfonyl)benzofuran* (**7g**). Following the preparation protocol of compound **7a**, starting from compound **5g** (160 mg, 0.57 mmol), the title compound **7g** was obtained as white solid (177 mg, 99%); m.p. 178–179.5 °C; ^1^H-NMR (400 MHz, CDCl_3_) δ (ppm): 7.66–7.57 (m, 3H), 7.35 (d, *J* = 8.4 Hz, 2H), 7.31–7.28 (m, 2H), 7.16 (td, *J*_1_ = 8.8 Hz, *J*_2_ = 2.0 Hz, 1H), 4.58 (s, 2H) (Supplementary Figure S31). ^13^C-NMR (151 MHz, CDCl_3_) δ (ppm): 163.12 (d, *J* = 248.7 Hz), 156.39 (d, *J* = 13.7 Hz), 149.01, 132.51, 132.15, 131.45, 124.27 (d, *J* = 10.4 Hz), 121.79, 117.98, 115.80, 114.00 (d, *J* = 24.5 Hz), 113.33, 100.18 (d, *J* = 27.0 Hz), 61.03 (Supplementary Figure S32). HR-MS (ESI) Calcd. for C_16_H_10_FNNaO_3_S [M + Na]^+^: 338.0263, Found: 338.0258.

*6-Fluoro-2-((4-bromobenzyl)sulfonyl)benzofuran* (**7h**). Following the preparation protocol of compound **7a**, starting from compound **5h** (188 mg, 0.56 mmol), the title compound **7h** was obtained as white solid (188 mg, 91%); m.p. 167–168 °C; ^1^H-NMR (400 MHz, CDCl_3_) δ (ppm): 7.62 (dd, *J*_1_ = 8.8 Hz, *J*_2_ = 5.6 Hz, 1H), 7.42 (d, *J* = 8.4 Hz, 2H), 7.33–7.27 (m, 2H), 7.15 (td, *J*_1_ = 9.2 Hz, *J*_2_ = 2.4 Hz, 1H), 7.10–7.04 (m, 2H), 4.48 (s, 2H) (Supplementary Figure S33). ^13^C-NMR (151 MHz, CDCl_3_) δ (ppm): 163.00 (d, *J* = 248.1 Hz), 156.35 (d, *J* = 13.8 Hz), 149.25, 132.22, 132.10, 125.98, 124.16 (d, *J* = 10.4 Hz), 123.74, 121.93, 115.59, 113.79 (d, *J* = 24.5 Hz), 100.06 (d, *J* = 26.9 Hz), 60.79 (Supplementary Figure S34). HR-MS (ESI) Calcd. for C_15_H_10_BrFNaO_3_S [M + Na]^+^: 390.9415, Found: 390.9411.

*6-Fluoro-2-((4-fluorobenzyl)sulfonyl)benzofuran* (**7i**). Following the preparation protocol of compound **7a**, starting from compound **5i** (400 mg, 1.44 mmol), the title compound **7i** was obtained as white solid (421 mg, 94%); m.p. 144–145 °C; ^1^H-NMR (400 MHz, CDCl_3_) δ (ppm): 7.61 (dd, *J*_1_ = 8.8 Hz, *J*_2_ = 5.2 Hz, 1H), 7.32–7.25 (m, 2H), 7.21–7.10 (m, 3H), 6.98 (t, *J* = 8.8 Hz, 2H), 4.50 (s, 2H) (Supplementary Figure S35). ^13^C-NMR (151 MHz, CDCl_3_) δ (ppm): 163.26 (d, *J* = 248.1 Hz), 162.96 (d, *J* = 247.8 Hz), 156.34 (d, *J* = 13.7 Hz), 149.36, 132.49 (d, *J* = 8.4 Hz), 124.10 (d, *J* = 10.4 Hz), 122.83 (d, *J* = 2.0 Hz), 121.96, 115.98 (d, *J* = 21.8 Hz), 115.48, 113.74 (d, *J* = 24.6 Hz), 100.04 (d, *J* = 26.9 Hz), 60.63 (Supplementary Figure S36). HR-MS (ESI) Calcd. for C_15_H_10_F_2_NaO_3_S [M + Na]^+^: 331.0216, Found: 331.0208.

*6-Fluoro-2-((4-methoxycarbonylbenzyl)sulfonyl)benzofuran* (**7j**). Following the preparation protocol of compound **7a**, starting from compound **5j** (290 mg, 0.92 mmol), the title compound **7j** was obtained as white solid (282 mg, 88%); m.p. 160–161 °C; ^1^H-NMR (400 MHz, CDCl_3_) δ (ppm): 7.95 (d, *J* = 8.4 Hz, 2H), 7.60 (dd, *J*_1_ = 8.8 Hz, *J*_2_ = 5.2 Hz, 1H), 7.32–7.23 (m, 4H), 7.14 (td, *J*_1_ = 9.2 Hz, *J*_2_ = 2.0 Hz, 1H), 4.58 (s, 2H), 3.90 (s, 3H) (Supplementary Figure S37). ^13^C-NMR (151 MHz, CDCl_3_) δ (ppm): 166.34, 163.01 (d, *J* = 248.1 Hz), 156.34 (d, *J* = 13.7 Hz), 149.17, 131.86, 130.91, 130.72, 129.98, 124.16 (d, *J* = 10.4 Hz), 121.88, 115.63, 113.80 (d, *J* = 24.6 Hz), 100.07 (d, *J* = 27.0 Hz), 61.18, 52.28 (Supplementary Figure S38). HR-MS (ESI) Calcd. for C_17_H_14_FO_5_S [M + H]^+^: 349.0546, Found: 349.0539.

**Note:** Except for target compounds, the synthesis and characterization of all other compounds mentioned in the Scheme 1 were described in the supporting information.

### 3.4. Biological Evaluation

#### 3.4.1. Expression and Purification of SIRT1−3

Human Sirtuin 1 (GenBank accession no. NM_012238), full length; human Sirtuin 2 (GenBank accession no. NM_012237), full length; human SIRT3 (GenBank accession no. NM_012239), 118−399. These three genes were cloned into pET21a (+). The recombinant proteins with an *N*-terminal fusion His_6_ tag were induced by IPTG (Isopropyl β-d-1-thiogalactopyranoside) and expressed in *Escherichia coli* BL21 (DE3) cells. The purification was performed by using a Qiagen Ni-NTA column (Duesseldorf, Germany). An SIRT1/2/3 enzymatic assay was performed according to the reference. In brief, the SIRT activity assay was performed using substrate peptide Ac-Arg-His-Lys-[Lys-(Ac)]-AMC. Costar^®^ EIA/RIA plate (Corning Incorporated, Corning, NY, USA, #3693) was used in this assay. 

#### 3.4.2. SIRT1-3 Assay

The stand control compounds and target compounds were tested against human recombinant SIRT1−3 using AMC-tagged deacetylation substrates.

The assay procedure involves two steps [23]. In the first step, the inhibition assay was run in 60 µL assay buffer (25 mM Tris-HCl, pH 8.0, 137 mM NaCl, 2.7 mM KCl, and 1 mM MgCl_2_ and 1 mg/mL BSA (Bovine albumin), 500 μM NADH (Nicotinamide adenine dinucleotide), 50 μM peptide substrate) and 1.0 µg recombinant SIRT protein. The assay was incubated at 37 °C for 2 h. Then, a 60 µL second batch assay buffer (50 mM Tris-HCl, pH 8.0, 100 mM NaCl, containing 10 µL trypsin (60,000 BAEE/mL) and 2 µL SIRT inhibitor nicotinamide (120 mM) were added. The reaction wells were mixed and incubated for another 37 °C for 20 min. The fluorescence was measured on a fluorometric reader (PerkinElmer EnSpire, Waltham, MA, USA) with excitation at 355 nm and emission at 460 nm. The IC_50_ calculation was calculated using GraphPad Prism Software (GraphPad Prism 5, Manufacturer, La Jolla, CA, USA). For each concentration, at least two wells were performed to calculate the average parameter.

## 4. Conclusions

In conclusion, we have discovered a series of novel benzofuran derivatives containing benzyl sulfoxide or benzyl sulfone scaffold. The enzymatic assay was performed against SIRT1–3 and all of the target compounds displayed potent selective SIRT2 inhibitory activity with IC_50_ values at the micromolar level. The preliminary SAR and the binding features of the benzofuran derivatives were analyzed. It has been demonstrated that benzyl sulfone moiety could be useful in developing potent SIRT2 inhibitors, and the benzofuran core could serve as an appropriate scaffold for discovering structurally novel and drug-like SIRT2 inhibitors. These results will be the foundation for further developments to achieve more potent benzofuran derivatives as selective SIRT2 inhibitors.

## Figures and Tables

**Table 1:**
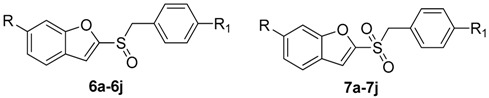
The chemical structures and inhibitory activities against SIRT1–3 (silent information regulator1–3) of compounds **6a**–**6j** and **7a**–**7j**.

Compd.	R	R_1_	IC50 (µM)		
			SIRT1	SIRT2	SIRT3
**6a**	CH_3_O	CH_3_O	>100	13.52	>100
**6b**	CH_3_O	CN	>100	15.14	>100
**6c**	CH_3_O	Br	>100	85.14	>100
**6d**	CH_3_O	F	>100	95.21	>100
**6e**	CH_3_O	CH_3_OCO	>100	15.68	>100
**6f**	F	CH_3_O	>100	27.89	>100
**6g**	F	CN	>100	32.75	>100
**6h**	F	Br	>100	74.93	>100
**6i**	F	F	>100	88.07	>100
**6j**	F	CH_3_OCO	>100	19.70	>100
**7a**	CH_3_O	CH_3_O	>100	7.66	>100
**7b**	CH_3_O	CN	>100	8.09	>100
**7c**	CH_3_O	Br	>100	17.76	>100
**7d**	CH_3_O	F	>100	43.93	>100
**7e**	CH_3_O	CH_3_OCO	>100	3.81	>100
**7f**	F	CH_3_O	>100	7.92	>100
**7g**	F	CN	>100	8.85	>100
**7h**	F	Br	>100	20.14	>100
**7i**	F	F	>100	51.42	>100
**7j**	F	CH_3_OCO	>100	6.16	>100
**Tenovin6**	-	-	37.50	15.32	89.31
**AGK2**	-	-	>100	1.56	52.80

## Supplementary Materials

The Supplementary Materials are available online.
