# Supplementary material for: Synthesis and Evaluation of Novel Benzofuran Derivatives as Selective SIRT2 Inhibitors
Source: Molecules. 2017 Aug 14;22(8):1348. doi: 10.3390/molecules22081348 (PMC6152376; doi:10.3390/molecules22081348)
Supplement: Supplementary file 1 [file molecules-22-01348-s001.pdf]

Supplementary data

Synthesis and evaluation of novel benzofuran derivatives as selective SIRT2 inhibitors

Yumei Zhou[1,2,3,], Huaqing Cui[3], Xiaoming Yu[3], Tao Peng[2], Gang Wang[2], Xiaoxue Wen[2], Yunbo Sun[2], Shuchen Liu[2], Shouguo Zhang[2]*, Liming Hu [1]* and Lin Wang[1,2]*

[1] *College of Life Science and Bioengineering, Beijing University of Technology, Beijing, 100124, China*

[2] *Institute of Radiation Medicine, Academy of Military Medical Sciences, Beijing 100850, China*

[3] *Institute of Materia Medica, Chinese Academy of Medical Sciences＆Peking Union Medical College, Beijing, 100050, China*

*Corresponding author Email: *wanglin@bmi.ac.cn; huliming@bjut.edu.cn ;zhangsg@bmi.ac.cn*

Contents

Synthesis of all intermediate compounds S2–S5

Representative NMR spectra S6–S25

1. Synthesis of all intermediate compounds

1.1. General procedure for condensation to generate imine **(2a-b)**:

A stirred solution of compound 2-Hydroxy-4-substitutephenylethanone, ethoxycarbonylhydrazine(1.1 equivalents) and concentrated HCl (0.08 equivalents) in ethanol was boiled for 2 h(monitoring by TLC following the consumption of the initial compound) and left at RT overnight .The separated precipitate was filtered off, washed with ethanol, and dried in air.

1.1.1. 2-Hydroxy-4-methoxyacetophenone ethoxycarbonylhydrazone **(2a)**: Intermediate 2**a** was synthesized by following above mentioned procedure for compound 1-(2-hydroxy-4-methoxyphenyl)ethanone (2.50g, 15.06 mmol) with ethoxycarbonylhydrazine (1.73g, 16.64 mmol) using concentrated HCl (0.10 mL, 1.20 mmol) to afford the pure product as white crystals (3.66 g, 96 %); mp 174-175 ℃. **^{1}H NMR** (400 MHz, DMSO-d_6) δ (ppm): 13.17 (s, 1H), 10.61 (s, 1H), 7.45 (d, J = 8.8 Hz, 1H), 6.47 – 6.40 (m, 2H), 4.20 (q, J = 6.8 Hz, 2H), 3.75 (s, 3H), 2.26 (s, 3H), 1.27 (t, J = 7.2 Hz, 3H). HRMS (ESI) Calcd. for $C_{12}H_{17}O_4N_2$ $[M+H]^+$: 253.1183; Found: 253.1182.

1.1.2. 2-Hydroxy-4-fluoroacetophenone ethoxycarbonylhydrazone **(2b)**: Intermediate 2**b** was synthesized by following above mentioned procedure for compound 1-(2-hydroxy-4-fluorophenyl)ethanone (5.00 g, 32.47 mmol) with ethoxycarbonylhydrazine (3.71 g, 35.71 mmol) using concentrated HCl (0.15 mL, 1.80 mmol) to afford the pure product as white crystals (7.39 g, 95 %); mp 175-177 ℃. **^{1}H NMR** (400 MHz, $CDCl_3$) δ (ppm): 7.39 – 7.36 (m, 1H), 6.69 (dd, J = 10.8, 2.4 Hz, 1H), 6.58 (td, J = 8.8, 2.0 Hz, 1H), 4.34 (q, J = 6.8 Hz, 2H), 2.26 (s, 3H), 1.38 (t, J = 6.4 Hz, 3H).

1.2. General procedure for cyclization to generate 1,2,3-thiadiazole **(3a-b)**:

To a solution of compound **(2)** in the mixture of chloroform and DMF(5:1) was dropwise added thionyl chloride(3.2 equivalents). The reaction gradually started to release of heat. reaction mixture became clear.when the reaction was judged to be complete (monitoring by TLC following the consumption of the initial compound).On cooling to 20 ℃ the reaction mixture was diluted with 100 mL of ice water. The organic layer was separated, the aqueous solution was extracted with dichloromethane,the organic layer was dried over anhydrous sodium sulfate,filter and concentrated under reduced pressure, the resulting residue was purified by column chromatograph on silica gel using petroleum ether and ethyl acetate solvent system as eluent to get the pure product.

1.2.1. 4-(2-Hydroxy-4-methoxyphenyl)-1,2,3-thiadiazole**(3a)**: Intermediate 3**a** was synthesized by following above mentioned procedure for intermediate **2a** (4.4g, 17.46 mmol) and thionyl chloride (4.1 mL, 56.16 mmol) to afford the pure product as white crystals (3.33 g, 92 %); mp 113–132 ℃, **^{1}H NMR** (400 MHz, $CDCl_3$) *δ* (ppm): 8.63 (s, 1H), 7.53 (d, *J* = 8.8 Hz, 1H), 6.66 (d, *J* = 2.4 Hz, 1H), 6.58 (dd, *J* = 8.8, 2.4 Hz, 1H), 3.85 (s, 3H); HRMS (ESI) Calcd. for $C_9H_9O_2N_2S$ $[M+H]^+$: 209.0379; Found: 209.0379.

1.2.2. 4-(2-Hydroxy-4-fluorophenyl)-1,2,3-thiadiazole **(3b)**: Intermediate **3b** was synthesized by carrying out above reaction for the intermediate **2b** (5.2 g, 21.67 mmol) and thionyl chloride (4.8 mL, 66.07 mmol) to afford the pure product as white crystals (4.16 g, 98 %); mp 171–172 ℃, **^{1}H NMR** (400 MHz, $CDCl_3$) *δ* (ppm): 10.86(br s, 1H), 8.73 (s, 1H), 7.61 (dd, *J* = 8.8, 6.2 Hz, 1H), 6.85 (dd, *J* = 10.0, 2.4 Hz, 1H), 6.75 – 6.71 (m, 1H).

1.3. General procedure for S-Alkylation to generate thioethers **(5a-j)**:

The thiadiazole**(3)** (1 equiv) and anhydrous potassium carbonate (2 equiv) were dissolved in freshly distilled DMF. The reaction mixture was heated at 100 ℃ and ground for 10 min at stirring (monitoring by TLC following the consumption of the initial compound and by the end of nitrogen liberation). On cooling to 30 ℃ compound 4-substitute benzyl chloride(1.05 equiv) was added to the reaction mixture. The reaction mixture was stirred for 10 min at 30 ℃, then it was poured into 100 mL of water. the aqueous solution was extracted with dichloromethane(4 times),the organic layer was dried over anhydrous sodium sulfate,filter and concentrated under reduced pressure, the resulting residue was purified by column chromatograph on silica gel using petroleum ether and ethyl acetate solvent system as eluent to get the pure product **(5a-f)**.

1.3.1. 6-methoxy-2-((4-methoxybenzyl)thio)benzo**[*b*]**furan **(5a)**: Intermediate **5a** was synthesized by following above procedure for the intermediate **3a** (0.624g, 3.0 mmol) ,anhydrous potassium carbonate(0.828g, 6.0 mmol) ,and 4-methoxybenzyl chloride (0.491g, 3.15 mmol) to obtain the product as white crystals (0.827g, 92 %); mp 91-92 ℃; **^{1}H NMR** (400 MHz, $CDCl_3$) *δ* (ppm): 7.32 (d, *J* = 8.4 Hz, 1H), 7.12 (d, *J* = 8.4, 2H), 6.99 (s, 1H), 6.84 (dd, *J* = 8.8, 2.0 Hz, 1H), 6.79 (d, *J* = 8.8, 2H), 6.61 (s, 1H), 4.06 (s, 2H), 3.85 (s, 3H), 3.77 (s, 3H).

1.3.2. 6-methoxy-2-((4-cyanobenzyl)thio)benzo**[*b*]**furan **(5b)**: Intermediate **5b** was synthesized by following above procedure for the intermediate **3a** (0.728 g, 3.5 mmol) ,anhydrous potassium carbonate(0.97 g, 7.0 mmol) ,and 4-cyanobenzyl bromide (0.686 g, 3.5

mmol) to obtain the product as white crystals (0.993 g, 96%); mp 54-55 ℃; ^{1}H NMR (400 MHz, $CDCl_3$) δ (ppm): 7.53 (d, J = 8.4 Hz, 2H), 7.33 (d, J = 8.8 Hz, 1H), 7.29 – 7.23 (m, 2H), 6.96 (s, 1H), 6.85 (dd, J = 8.8, 2.4 Hz, 1H), 6.60 (d, J = 0.8 Hz, 1H), 4.07 (s, 2H), 3.85 (s, 3H).

1.3.3. 6-methoxy-2-((4-bromobenzyl)thio)benzo[***b***]furan **(5c)**: Intermediate **5c** was synthesized by following above procedure for the intermediate **3a** (0.728 g, 3.5 mmol) ,anhydrous potassium carbonate(0.96 g, 6.96 mmol) ,and 4-bromobenzyl bromide (0.88 g, 3.5 mmol) to obtain the product as white crystals (1.145 g, 94%); mp 49-50 ℃; ^{1}H NMR (400 MHz, $CDCl_3$) δ (ppm): 7.37 (d, J = 8.4 Hz, 2H), 7.33 (d, J = 8.8 Hz, 1H), 7.05 (d, J = 8.0 Hz, 2H), 6.97 (s, 1H), 6.85 (dd, J_1 = 8.4, J_2 = 2.0 Hz, 1H), 6.61 (d, J = 0.8, 1H), 4.02 (s, 2H), 3.85 (s, 3H).

1.3.4. 6-methoxy-2-((4-fluorobenzyl)thio)benzo[***b***]furan **(5d)**: Intermediate **5d** was synthesized by following above procedure for the intermediate **3a** (0.624 g, 3.0 mmol) ,anhydrous potassium carbonate(0.828 g, 6.0 mmol),and 4-fluorobenzyl bromide (0.595 g, 3.15 mmol) to obtain the product as colourless oil (0.812 g, 94%); ^{1}H NMR (400 MHz, $CDCl_3$) δ (ppm): 7.32 (d, J = 8.4 Hz, 1H), 7.16 – 7.12 (m, 2H), 6.97 (s, 1H), 6.93 (t, J = 8.4, 2H), 6.84 (dd, J_1 = 8.8, J_1 = 2.0 Hz, 1H), 6.60 (s, 1H), 4.05 (s, 2H), 3.85 (s, 3H).

1.3.5. 6-methoxy-2-((4-methoxycarbonylbenzyl)thio)benzo[***b***]furan **(5e)**: Intermediate **5e** was synthesized by following above procedure for the intermediate **3a** (0.728 g, 3.5 mmol),anhydrous potassium carbonate(0.97 g, 7.0 mmol),and Methyl 4-(bromomethyl)benzoate (0.801 g, 3.5 mmol) to obtain the product as light white solid (1.099 g, 95%); mp 77-78 ℃; ^{1}H NMR (400 MHz, $CDCl_3$) δ (ppm): 7.92 (d, J = 8.0 Hz, 2H), 7.31 (d, J = 8.4 Hz, 1H), 7.25 – 7.22 (m, 2H), 6.97 (d, J = 1.2 Hz, 1H), 6.84 (dd, J = 8.4, 2.0 Hz, 1H), 6.59 (s, 1H), 4.10 (s, 2H), 3.89 (s, 3H), 3.85 (s, 3H).

1.3.6. 6-fluoro-2-((4-methoxybenzyl)thio)benzo[***b***]furan **(5f)**: Intermediate **5f** was synthesized by following above procedure for the intermediate **3b** (0.882 g, 4.5 mmol),anhydrous potassium carbonate(1.24 g, 9.0 mmol),and 4-methoxybenzyl bromide (0.737 g, 4.72 mmol) to obtain the product as Light-yellow crystals (1.199 g, 93%); mp 78-79 ℃; ^{1}H NMR (400 MHz, $CDCl_3$) δ (ppm): 7.37 (dd, J = 8.8, 5.6 Hz, 1H), 7.17 (dd, J = 8.8, 1.6 Hz, 1H), 7.13 (d, J = 8.8, 2H), 7.00 – 6.95 (m, 1H), 6.82 – 6.77 (m, 2H), 6.63 (s, 1H), 4.09 (s, 2H), 3.77 (s, 3H).

1.3.7. 6-fluoro-2-((4-cyanobenzyl)thio)benzo**[*b*]**furan **(5g)**: Intermediate **5g** was synthesized by following above procedure for the intermediate **3b** (0.882 g, 4.5 mmol),anhydrous potassium carbonate(1.24 g, 9.0 mmol),and 4-cyanobenzyl bromide (0.926 g, 4.73 mmol) to obtain the product as white crystals (1.199 g, 94%); mp 85-86 ℃; ^{1}H NMR (400 MHz, $CDCl_3$) δ (ppm): 7.40 – 7.36 (m, 3H), 7.17 (dd, J_1 = 8.8, J_2 = 2.0 Hz, 1H), 7.06 (d, J = 8.4 2H), 7.01 – 6.95 (m, 1H), 6.64 (d, J = 0.8 Hz, 1H), 4.05 (s, 2H).

1.3.8. 6-fluoro-2-((4-bromobenzyl)thio)benzo**[*b*]**furan **(5h)**: Intermediate **5h** was synthesized by following above procedure for the intermediate **3b** (0.882 g, 4.5 mmol),anhydrous potassium carbonate(1.24 g, 9.0 mmol),and 4-bromobenzyl bromide (1.181 g, 4.73 mmol) to obtain the product as white crystals (1.423 g, 94%); mp 100-101.5 ℃; ^{1}H NMR (400 MHz, $CDCl_3$) δ (ppm): 7.54 (d, J = 8.0, 2H), 7.39 (dd, J_1 = 8.8, J_2 = 5.6 Hz, 1H), 7.27 (d, J = 8.0 2H), 7.16 (dd, J = 8.4, 1.2 Hz, 1H), 7.02 – 6.96 (m, 1H), 6.63 (d, J = 0.8 Hz, 1H), 4.12 (s, 2H).

1.3.9. 6-fluoro-2-((4-fluorobenzyl)thio)benzo**[*b*]**furan **(5i)**: Intermediate **5i** was synthesized by following above procedure for the intermediate **3b** (0.882 g, 4.5 mmol),anhydrous potassium carbonate(1.24 g, 9.0 mmol),and 4-fluorobenzyl bromide (0.893 g, 4.73 mmol) to obtain the product as white crystals (1.151 g, 93%); mp 55.5-57 ℃; ^{1}H NMR (400 MHz, $CDCl_3$) δ (ppm): 7.35 (dd, J_1 = 8.4, J_2 = 5.6 Hz, 1H), 7.17 – 7.12 (m, 3H), 7.00 – 6.88 (m, 3H), 6.61 (d, J = 0.8 Hz, 1H), 4.07 (s, 2H).

1.3.10. 6-fluoro-2-((4-methoxycarbonylbenzyl)thio)benzo**[*b*]**furan **(5j)**: Intermediate **5j** was synthesized by following above procedure for the intermediate **3b** (0.882 g, 4.5 mmol),anhydrous potassium carbonate(1.24 g, 9.0 mmol),and Methyl 4-(bromomethyl)benzoate (1.082 g, 4.73 mmol) to obtain the product as white crystals (1.388 g, 97%); mp 91.5-93 ℃; ^{1}H NMR (400 MHz, $CDCl_3$) δ (ppm): 7.93 (d, J = 8.0, 2H), 7.37 (dd, J_1 = 8.4, J_2 = 5.2 Hz, 1H), 7.28 – 7.23 (m, 2H), 7.16 (dd, J_1 = 8.8, J_2 = 1.6 Hz, 1H), 7.00 – 6.95 (m, 1H), 6.62 (d, J = 0.8 Hz, 1H), 4.13 (s, 2H), 3.89 (s, 3H).

2. Representative NMR spectra

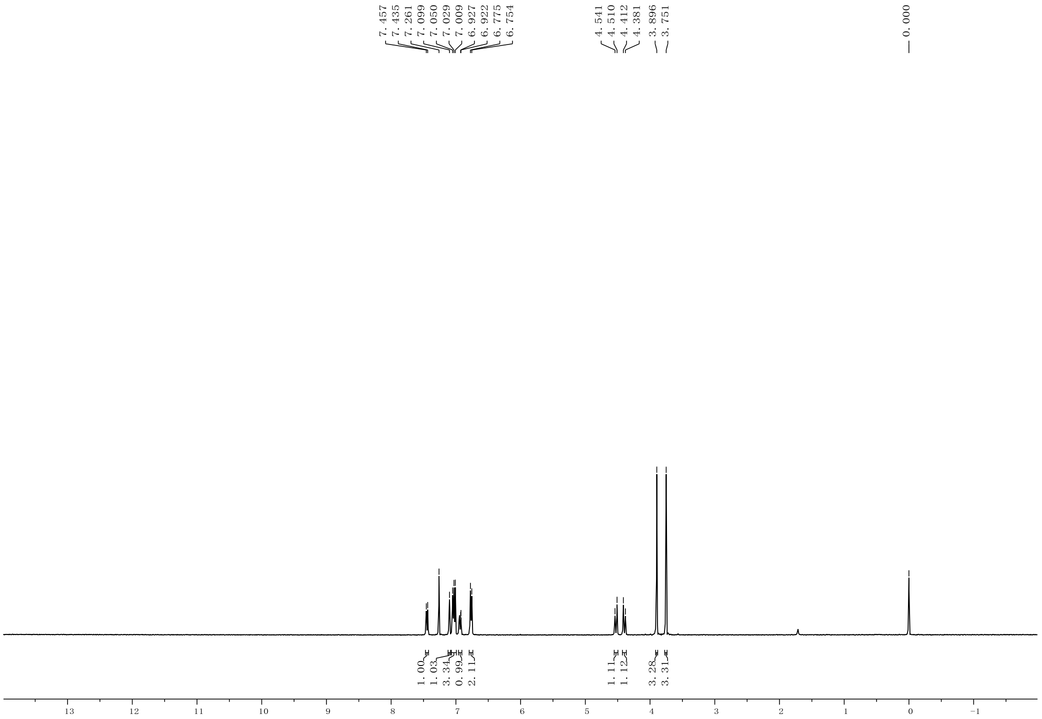

Figure S1. ^{1}H NMR spectrum of 6a (400 MHz, $CDCl_3$)

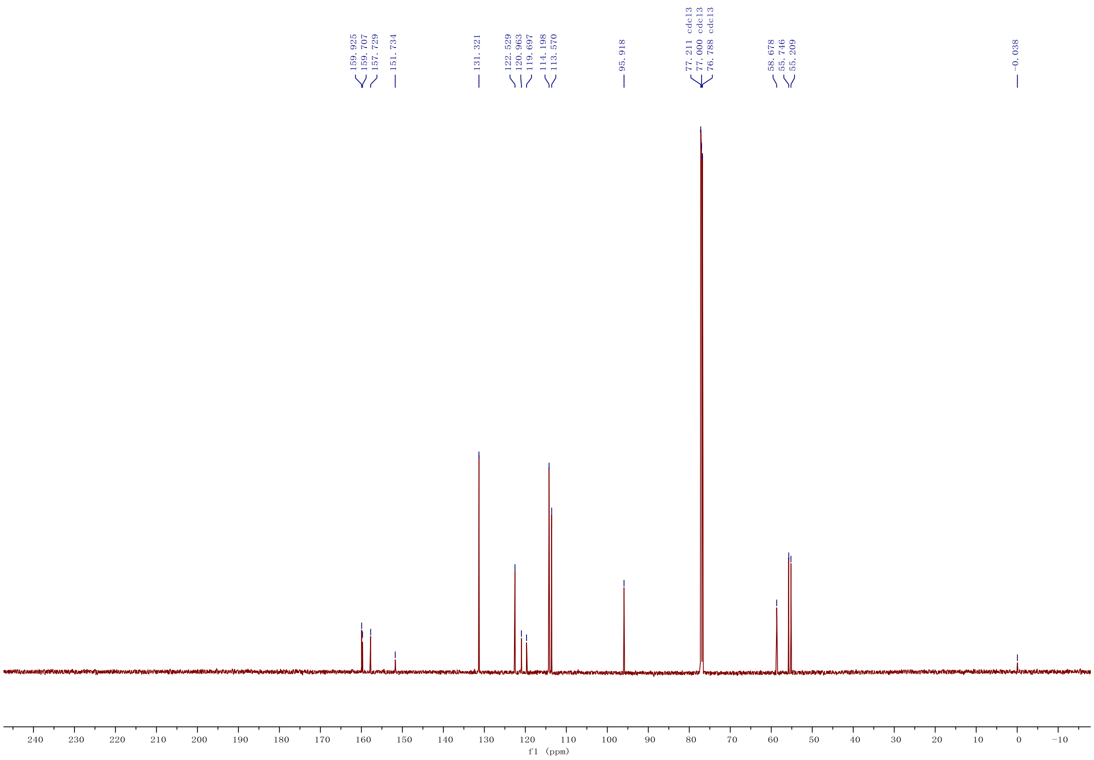

Figure S2. ^{13}C NMR spectrum of 6a.

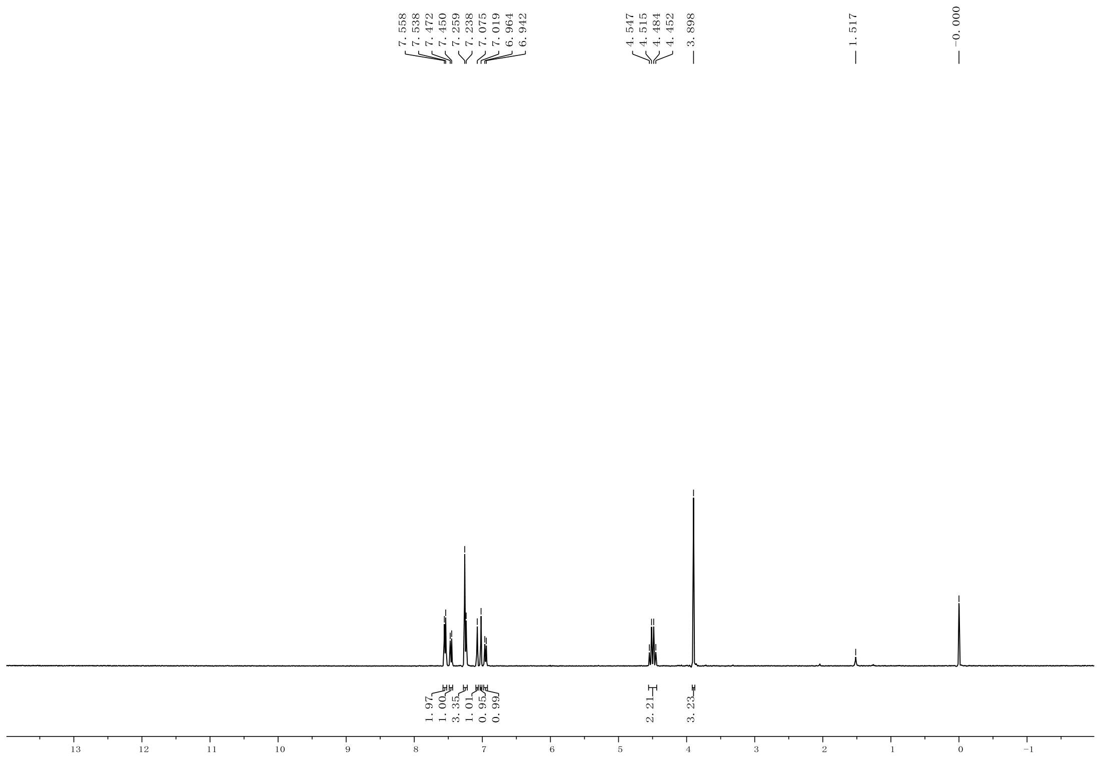

Figure S3. ^{1}H NMR spectrum of 6b (400 MHz, $CDCl_3$)

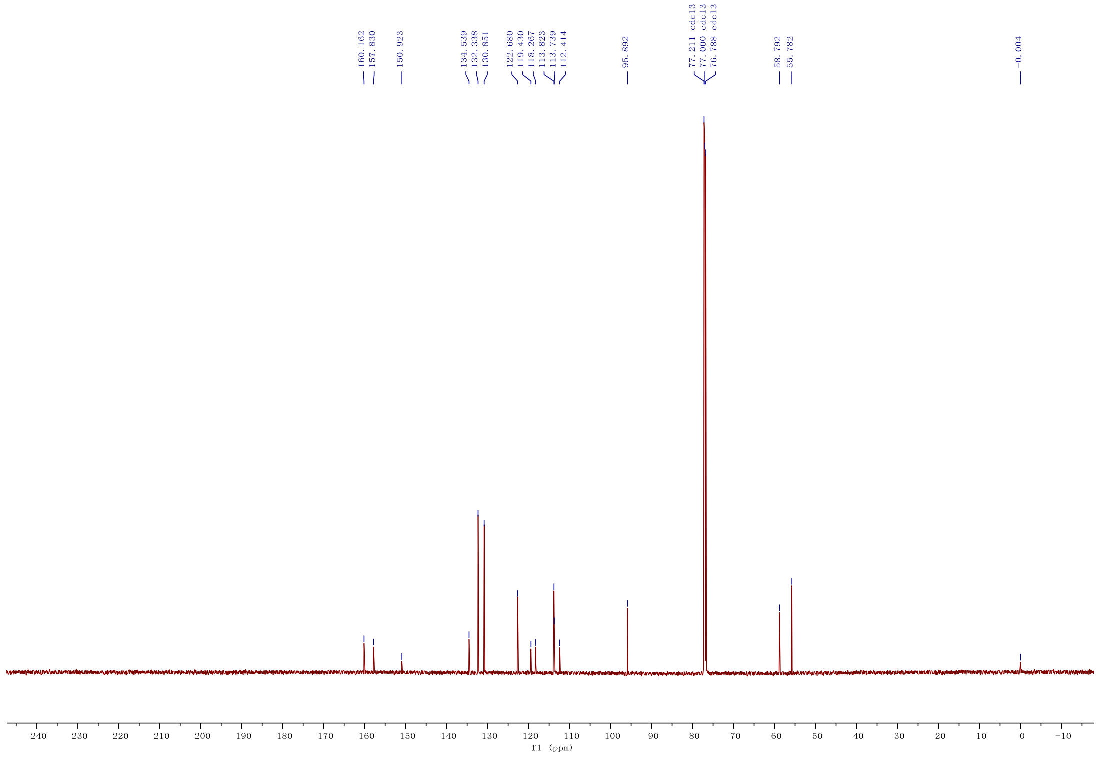

Figure S4. ^{13}C NMR spectrum of 6b.

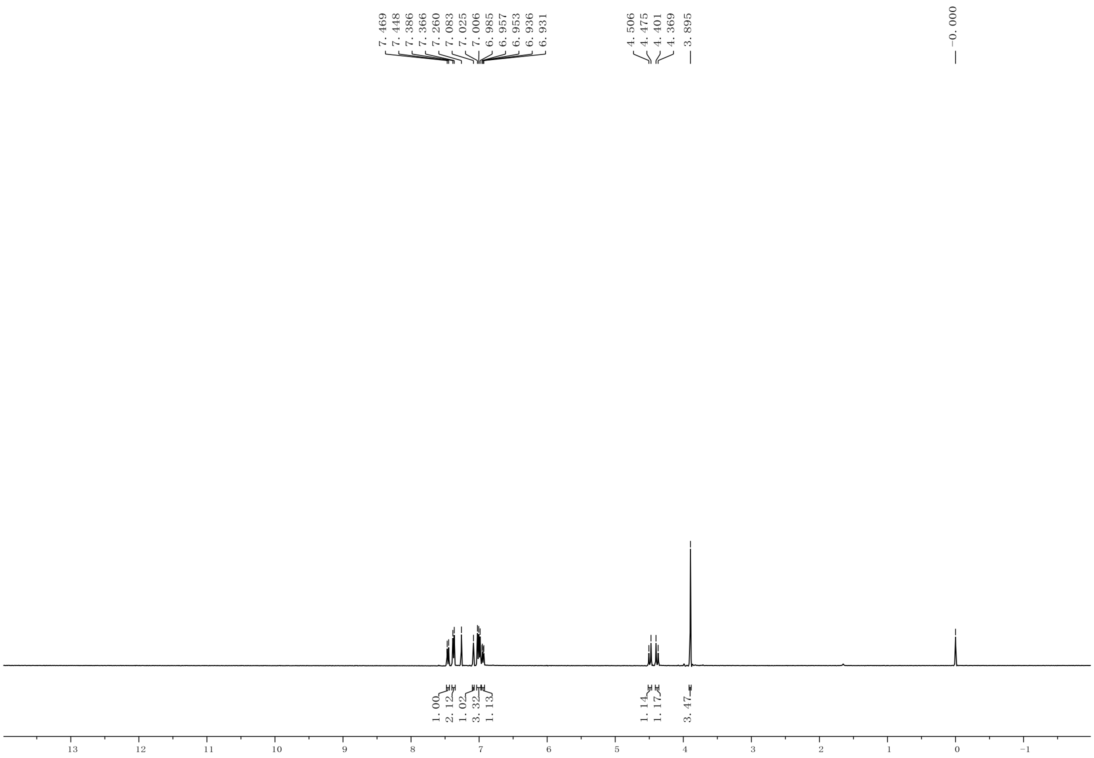

Figure S5. ^{1}H NMR spectrum of 6c

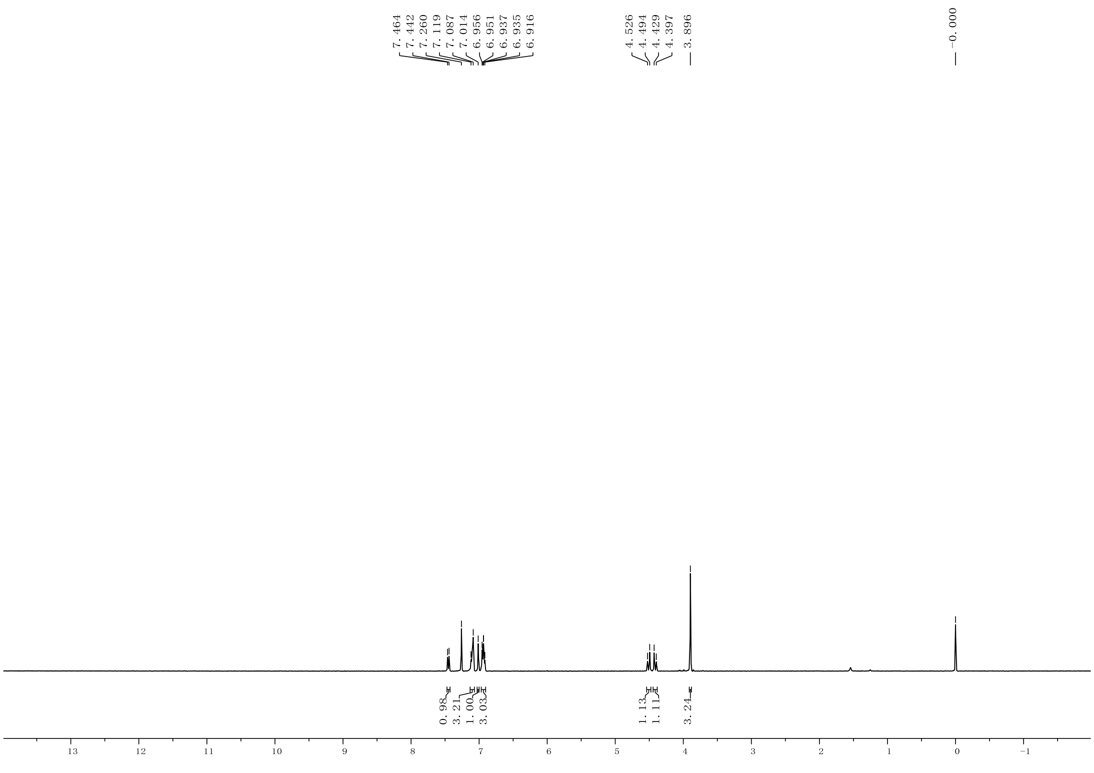

Figure S6. ^{1}H NMR spectrum of 6d

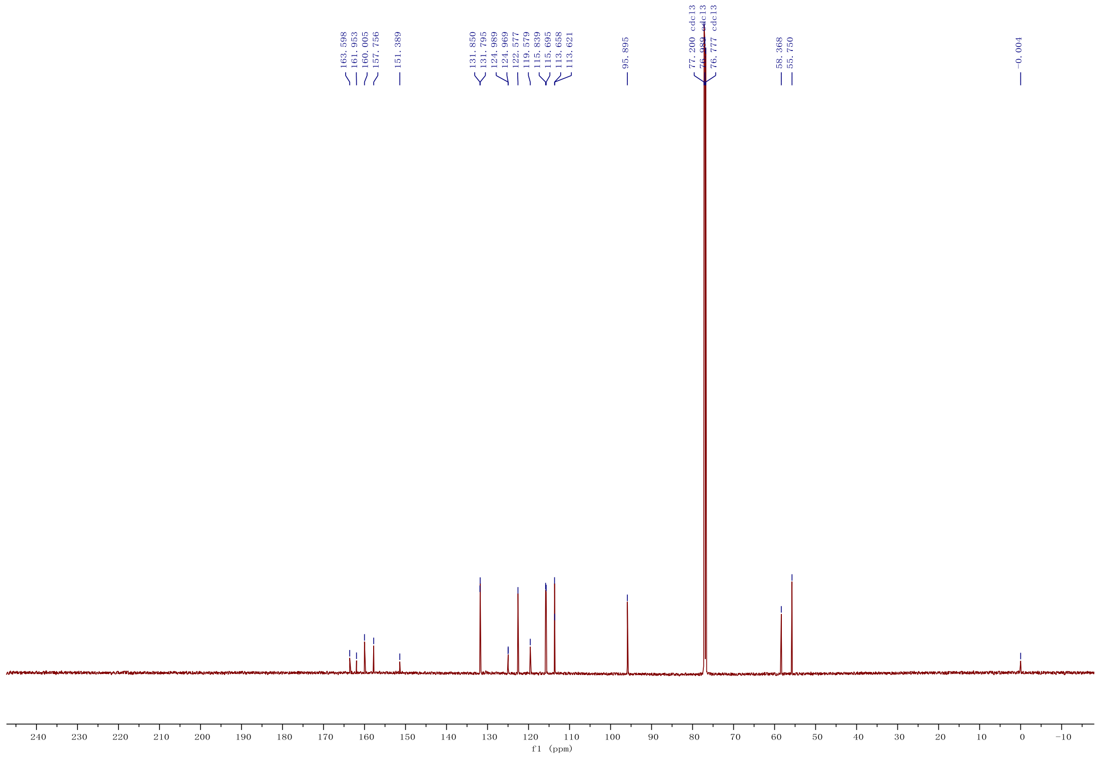

Figure S7. ^{13}C NMR spectrum of 6d.

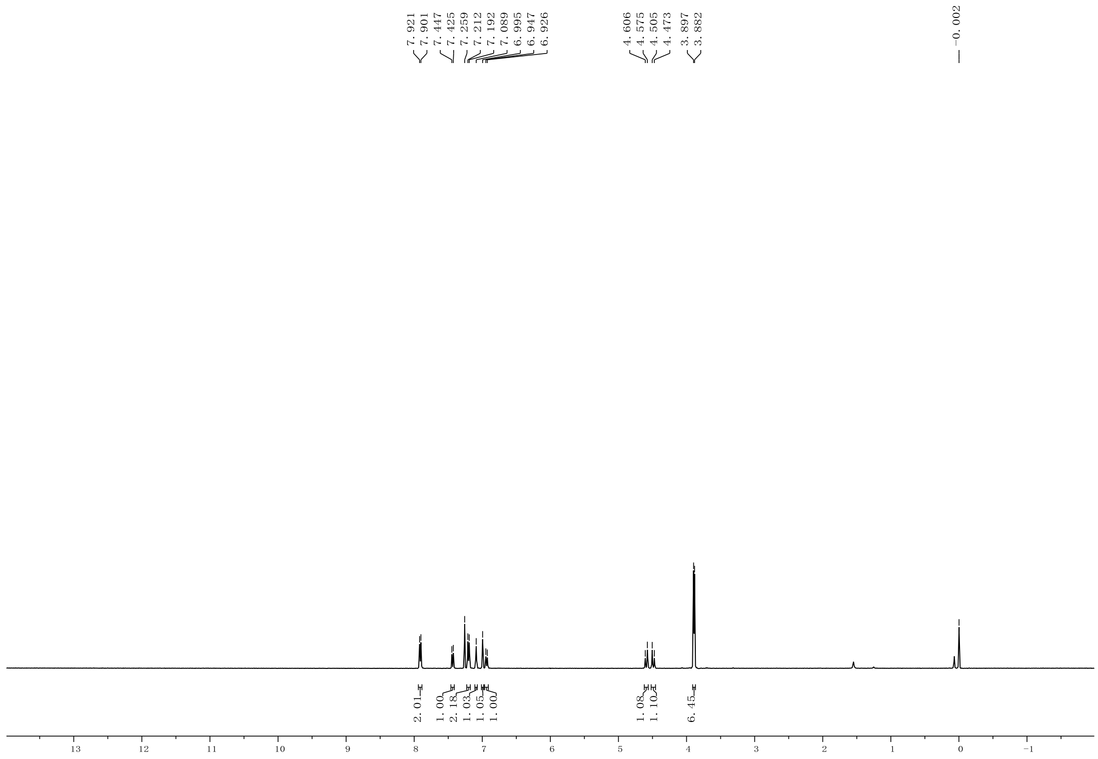

Figure S8. ^{1}H NMR spectrum of 6e.

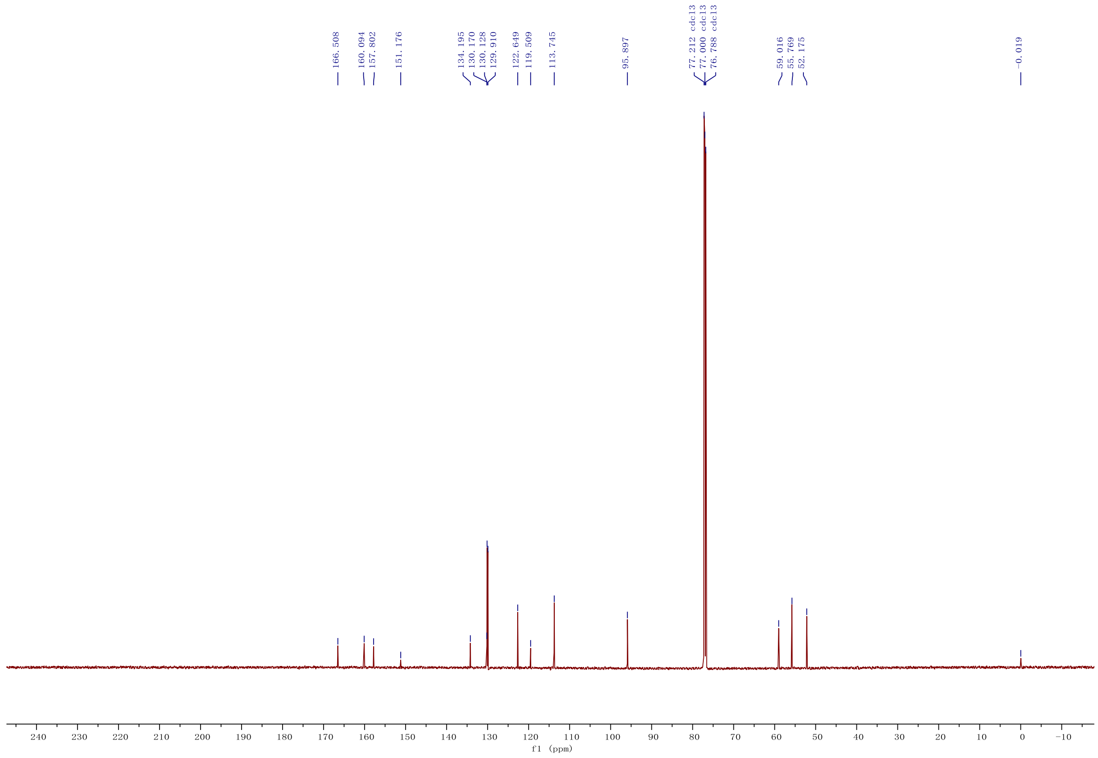

Figure S9. ^{13}C NMR spectrum of 6e.

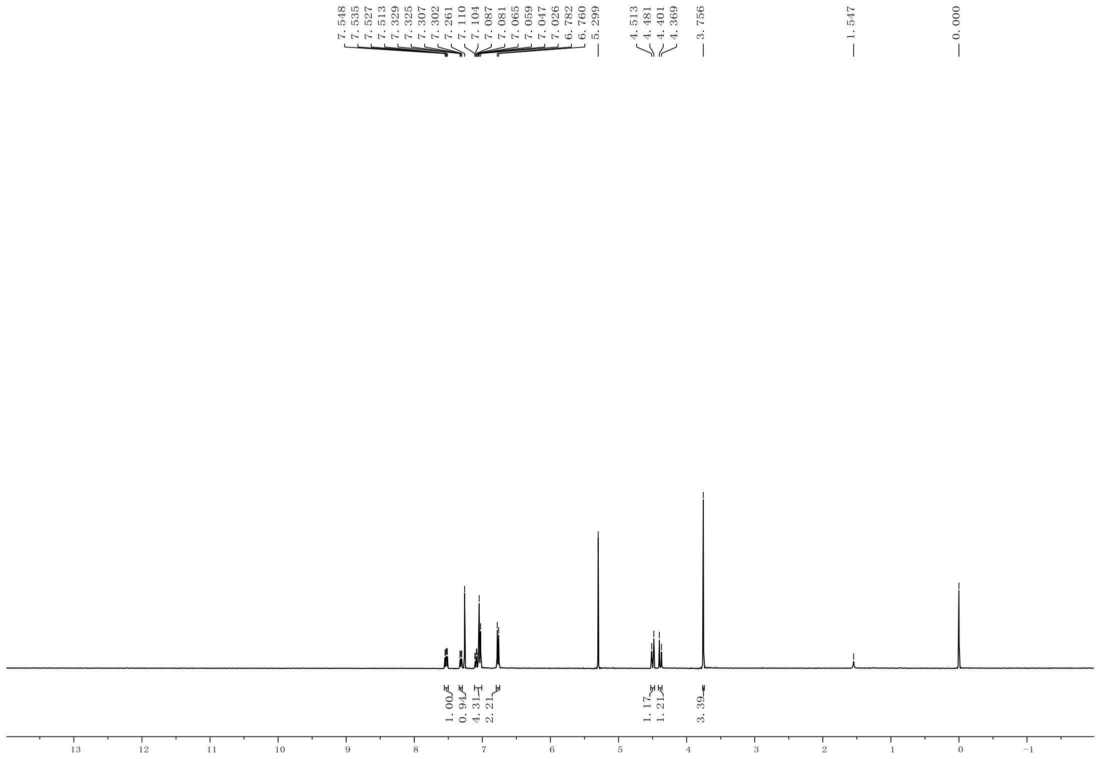

Figure S10. ^{1}H NMR spectrum of 6f.

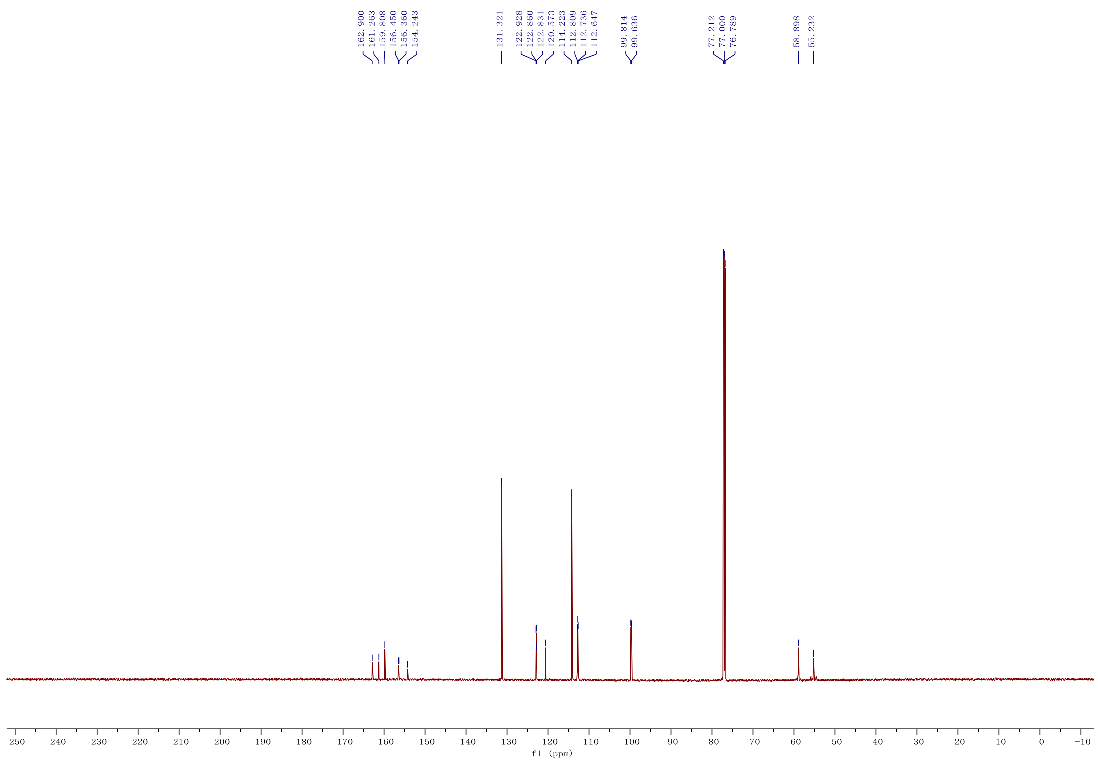

Figure S11. ^{13}C NMR spectrum of 6f.

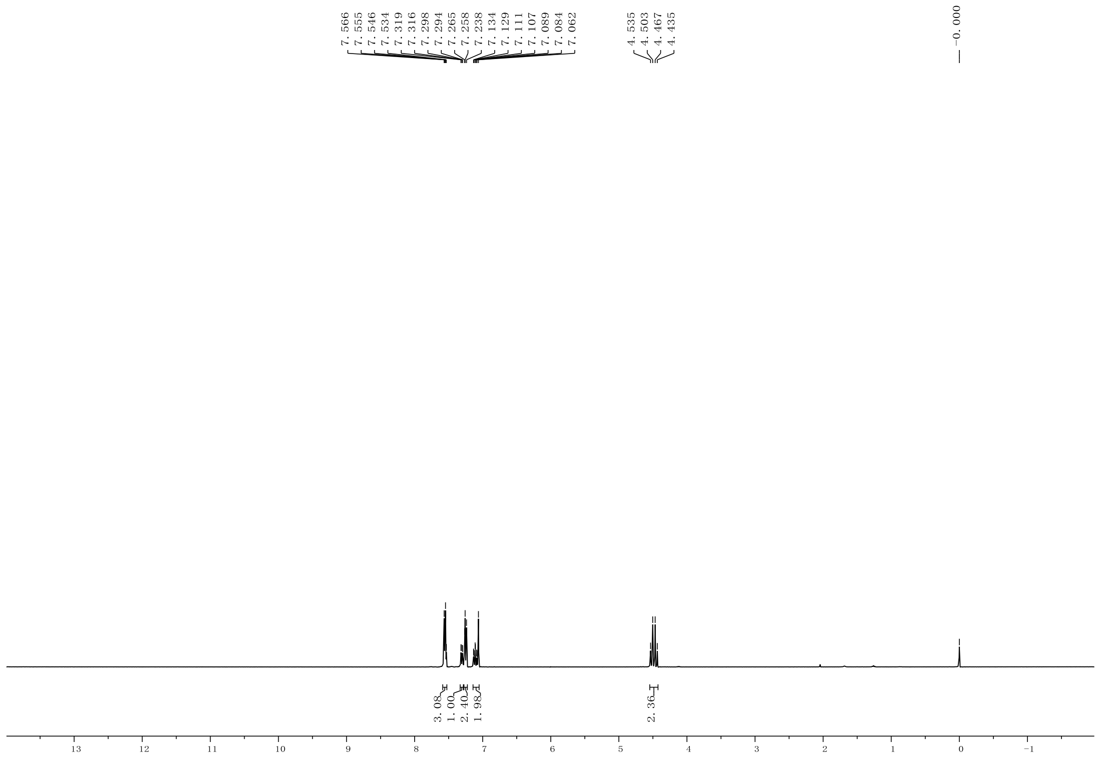

Figure S12. ^{1}H NMR spectrum of 6g.

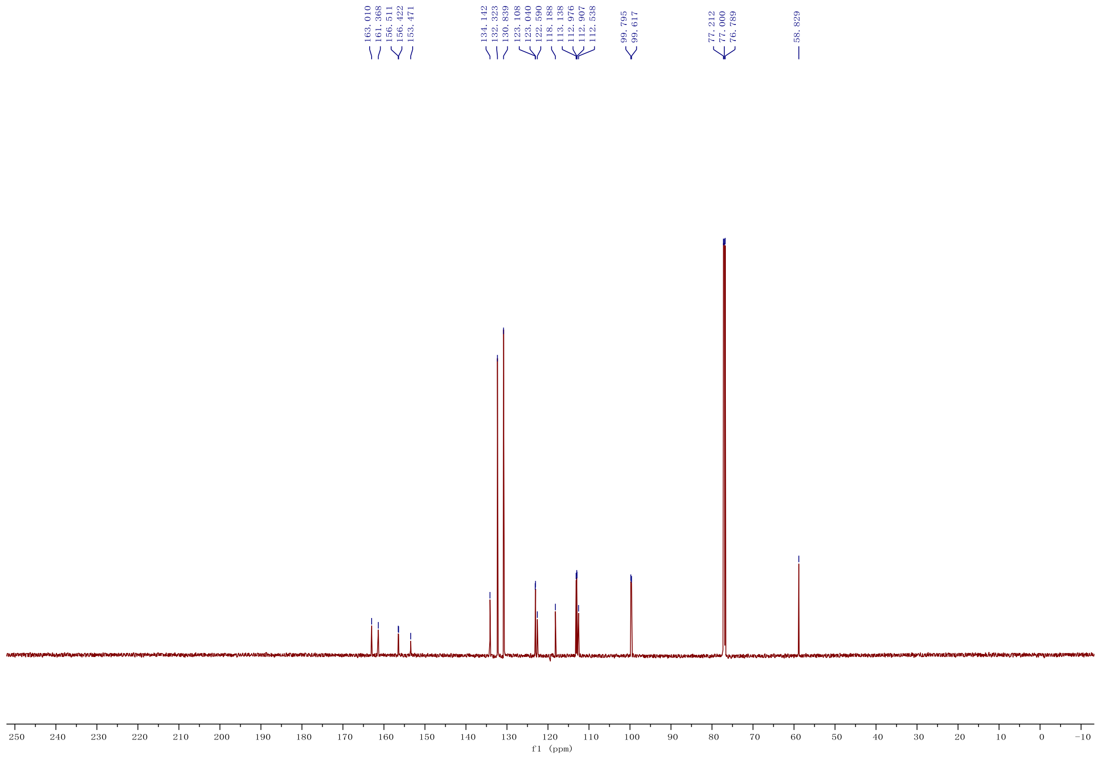

Figure S13. ^{13}C NMR spectrum of 6g.

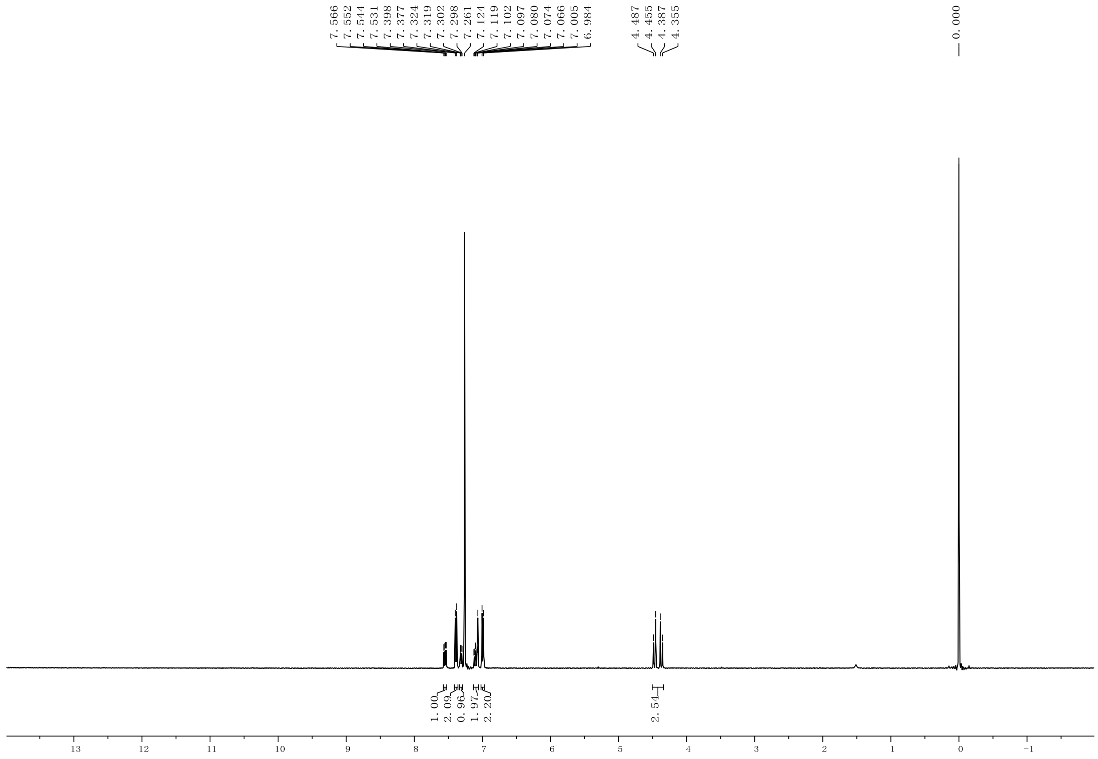

Figure S14. ^{1}H NMR spectrum of 6h.

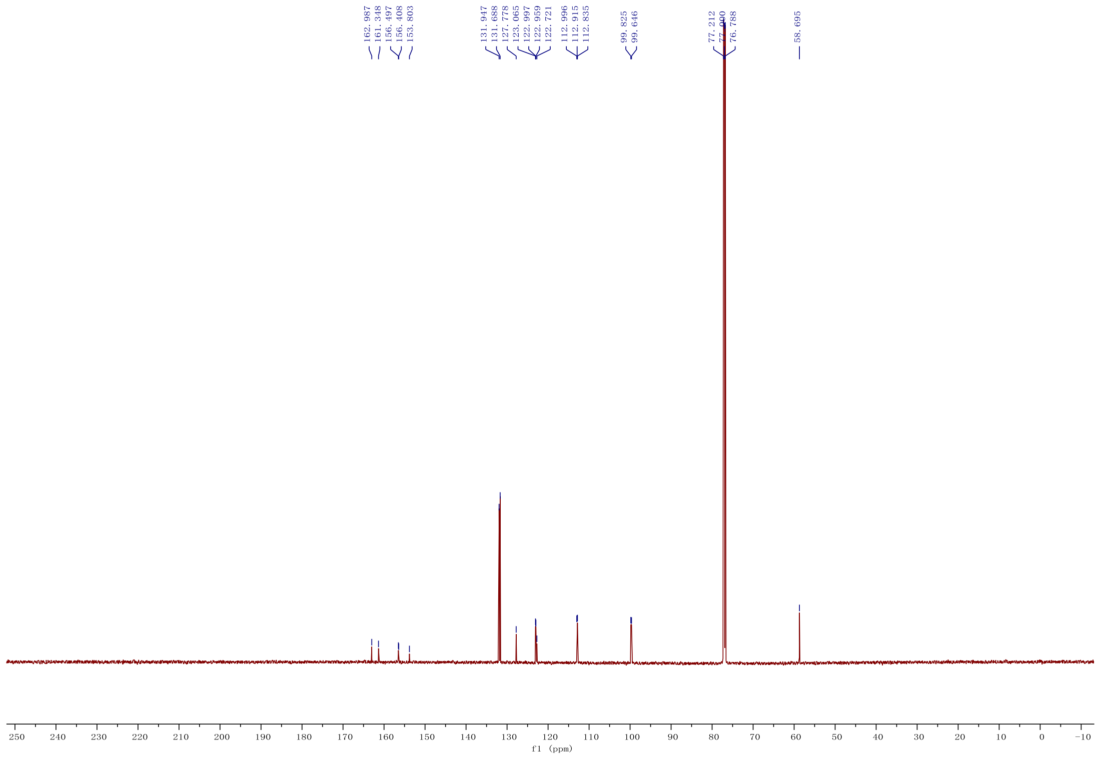

Figure S15. ^{13}C NMR spectrum of 6h.

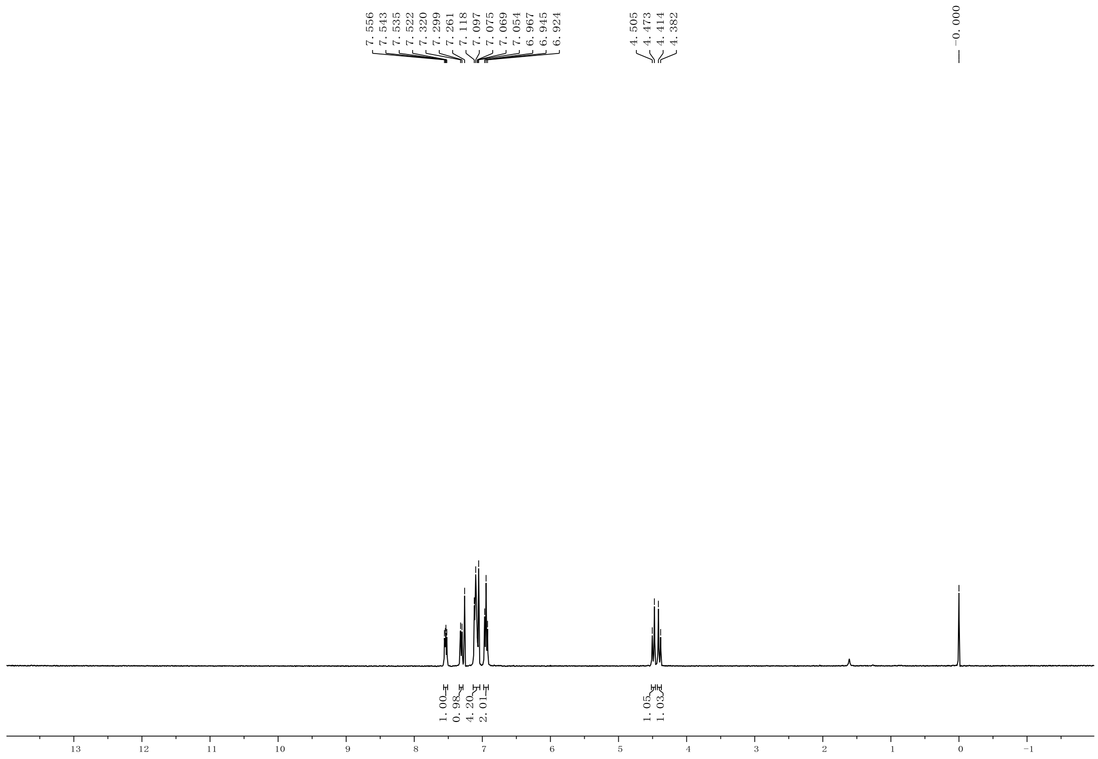

Figure S16. ^{1}H NMR spectrum of 6i.

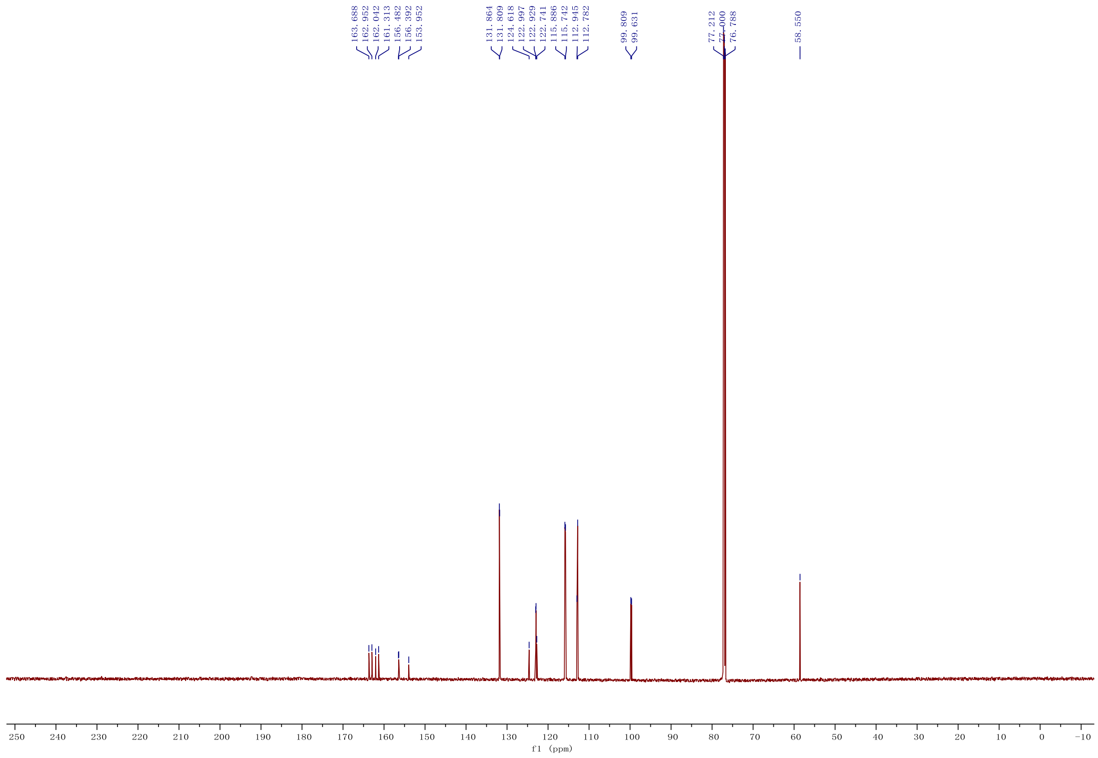

Figure S17. ^{13}C NMR spectrum of 6i.

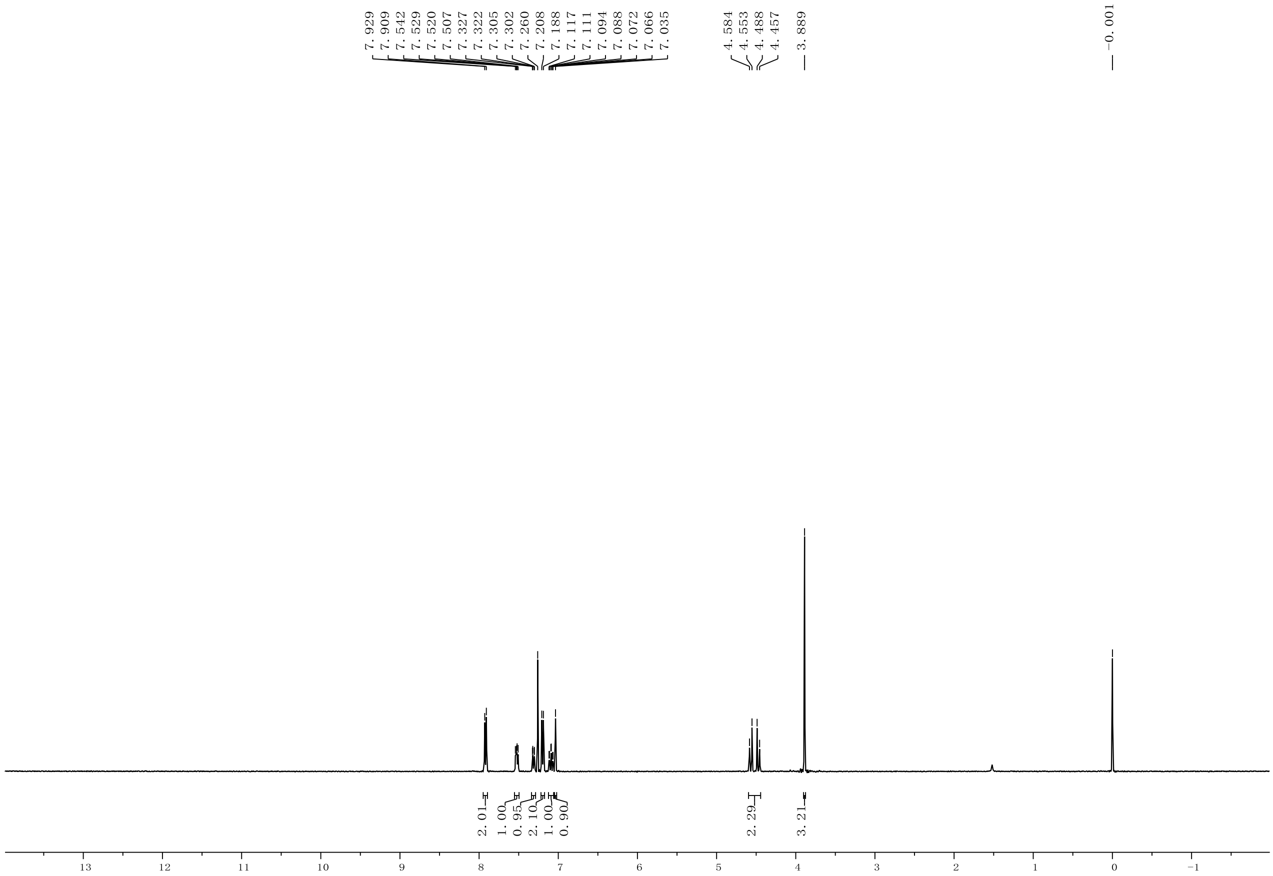

Figure S18. ^{1}H NMR spectrum of 6j.

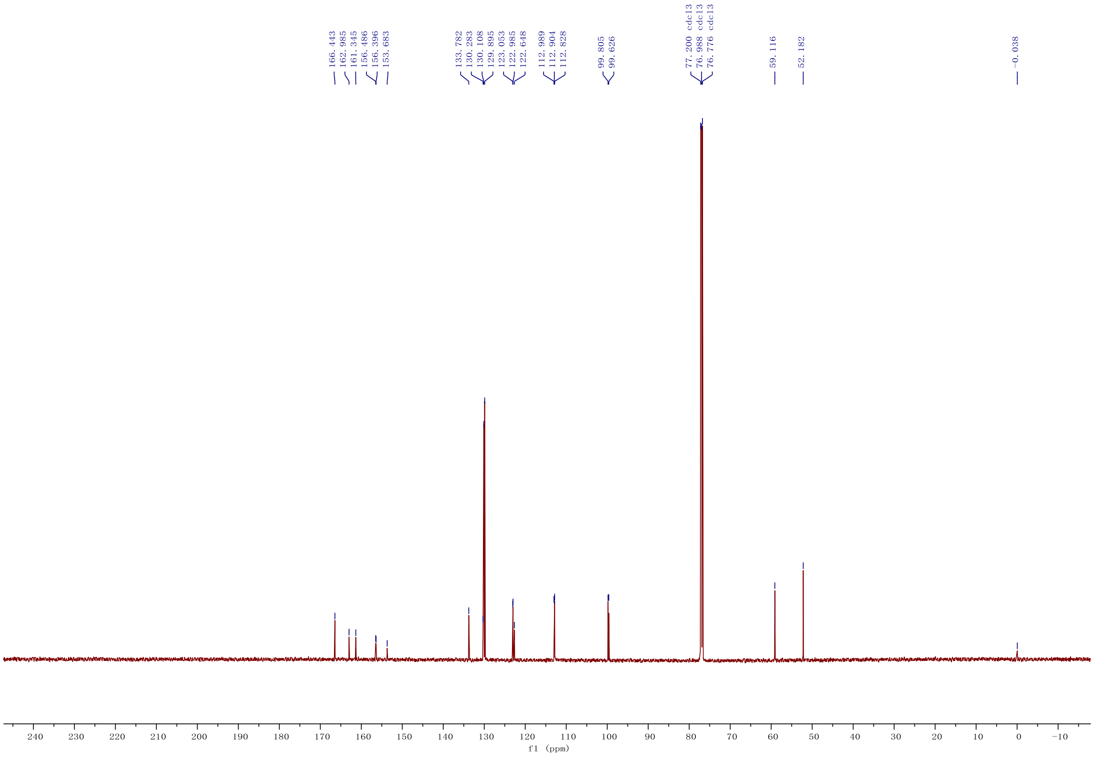

Figure S19. ^{13}C NMR spectrum of 6j.

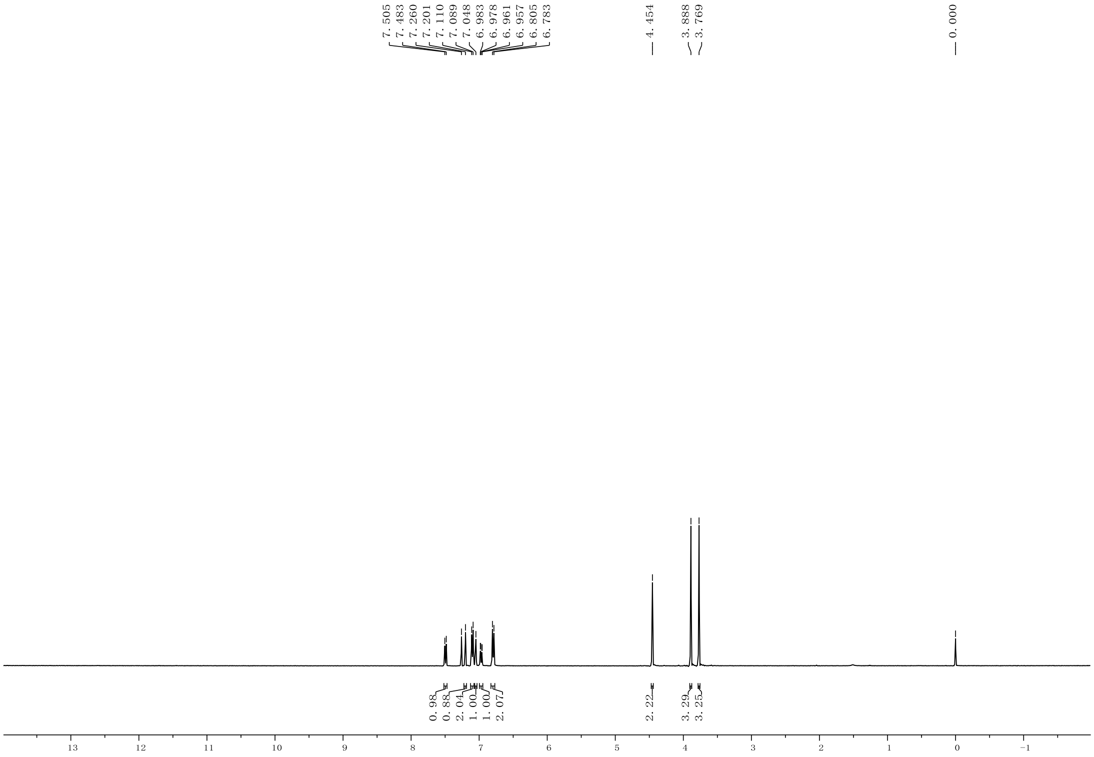

Figure S20. ^{1}H NMR spectrum of 7a.

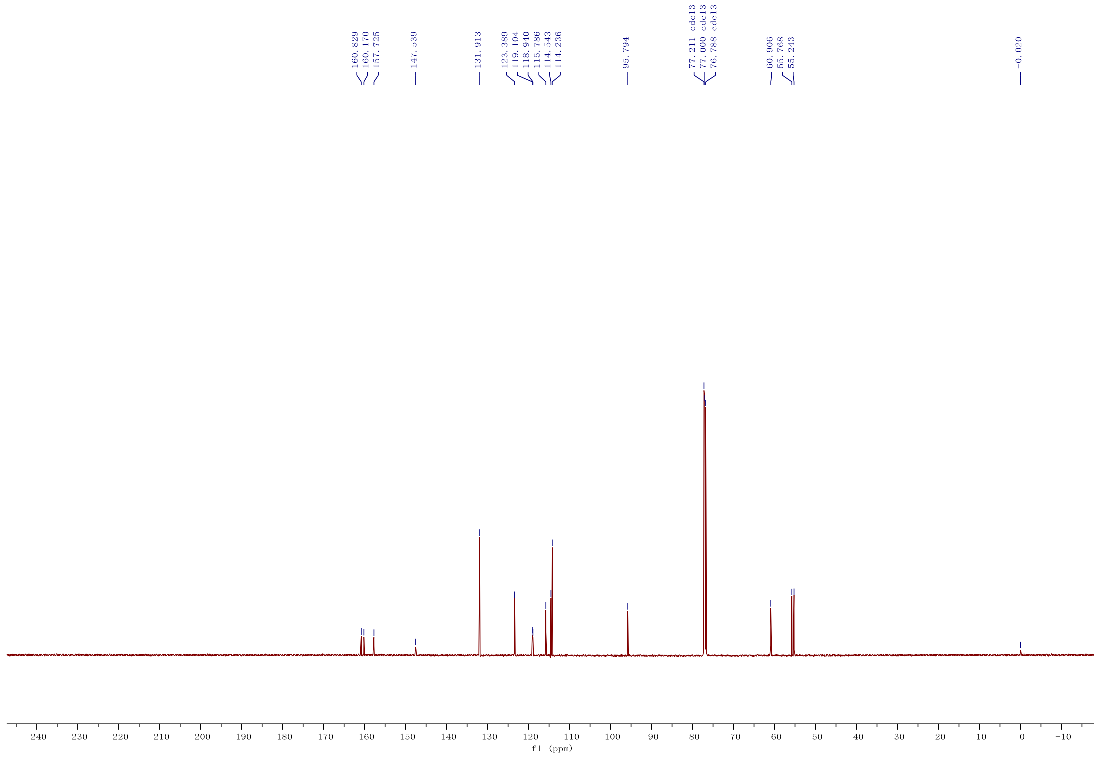

Figure S21. ^{13}C NMR spectrum of 7a.

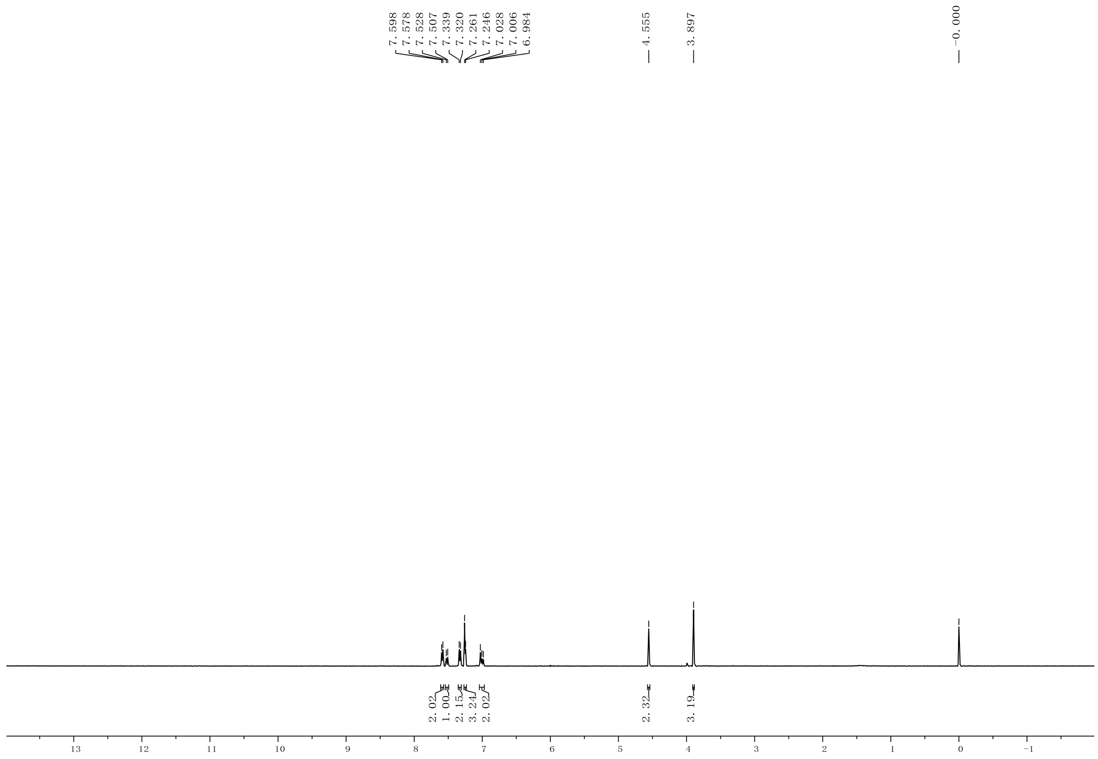

Figure S22. ^{1}H NMR spectrum of 7b.

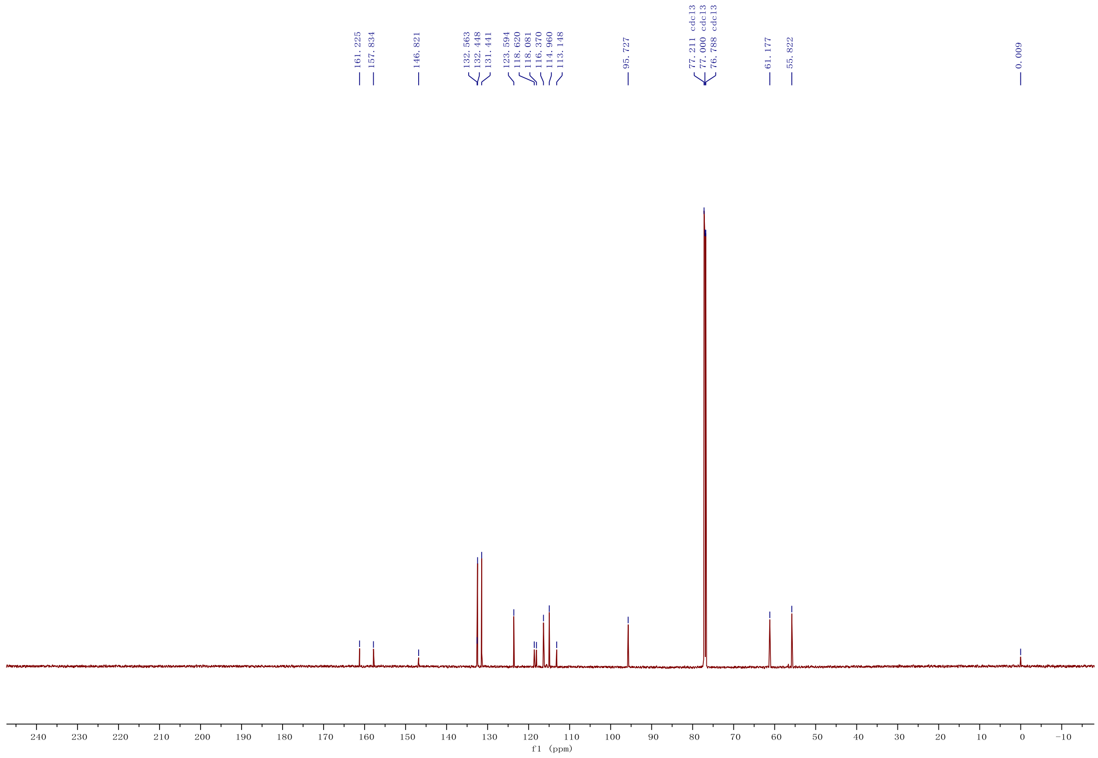

Figure S23. ^{13}C NMR spectrum of 7b.

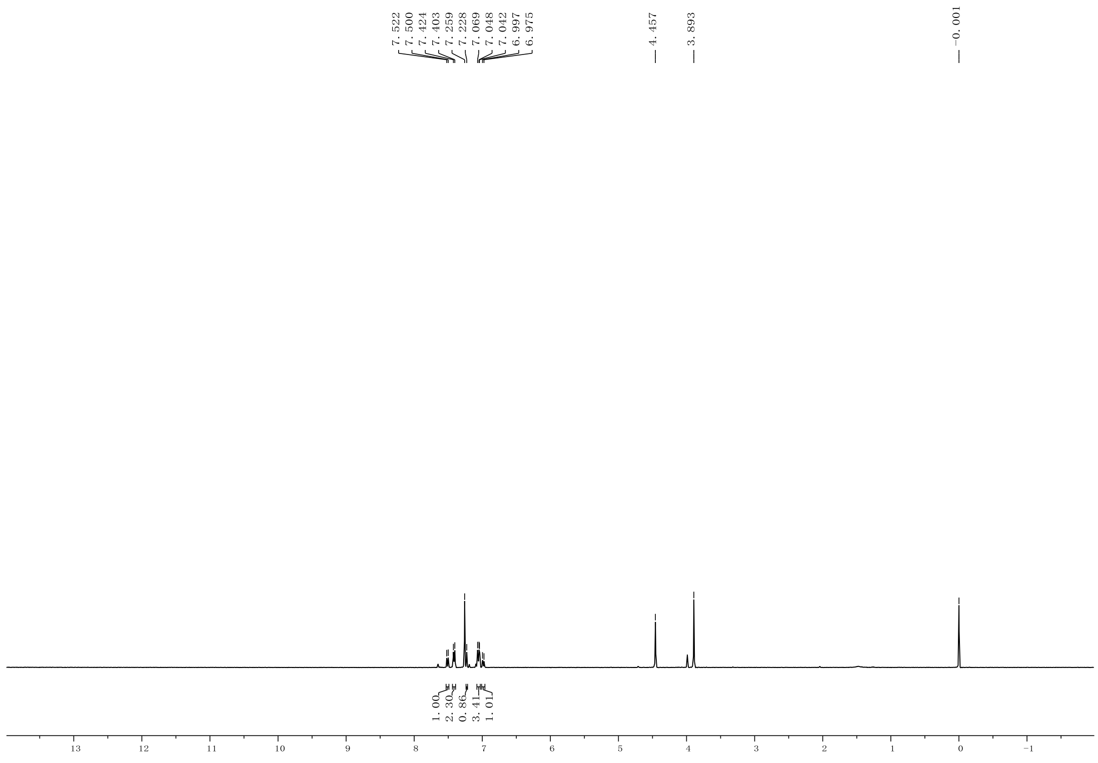

Figure S24. ^{1}H NMR spectrum of 7c.

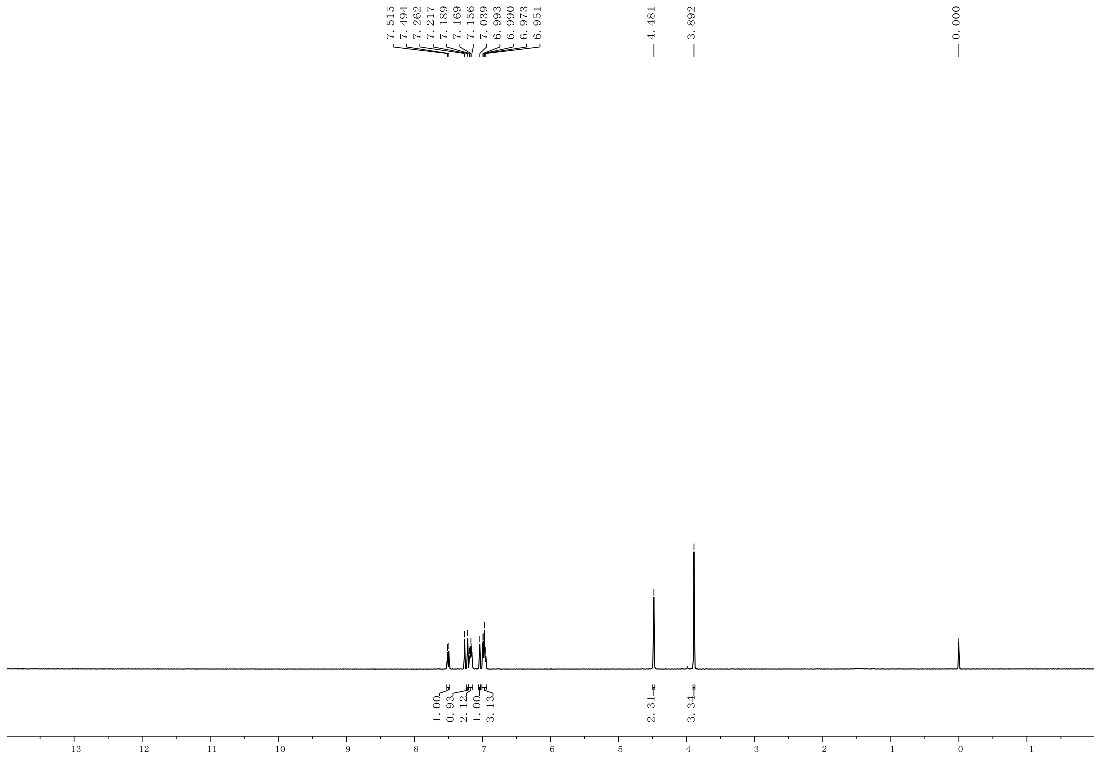

Figure S25. ^{1}H NMR spectrum of 7d

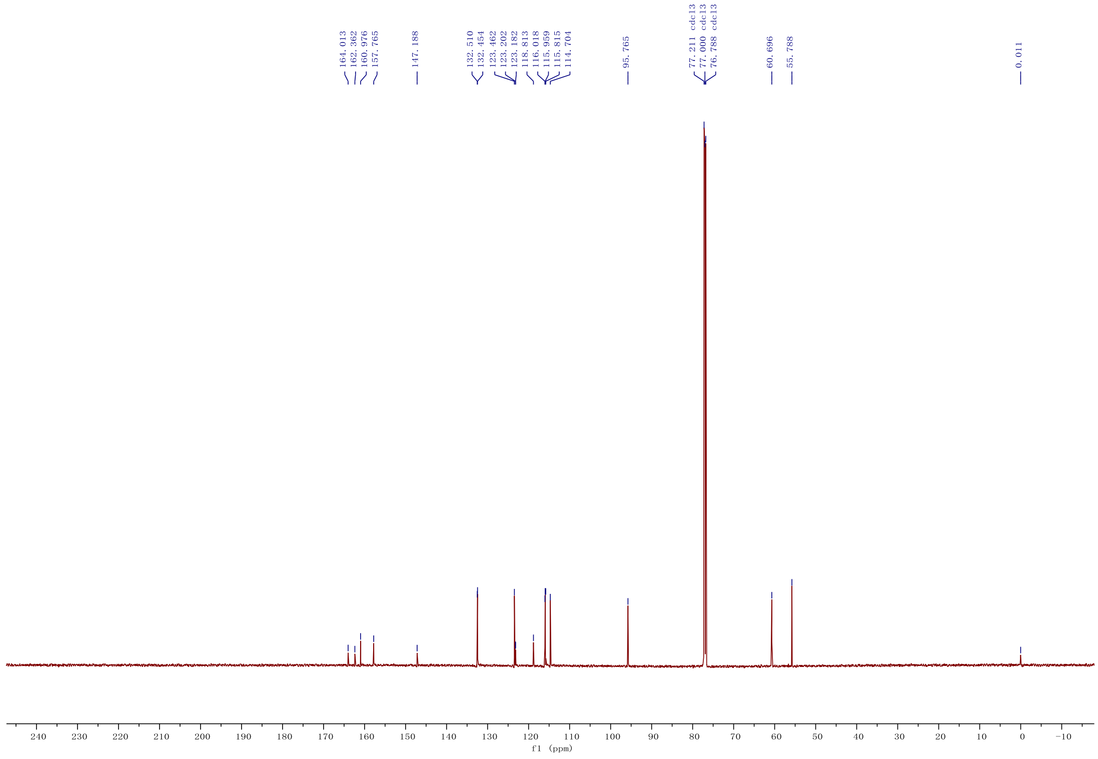

Figure S26. ^{13}C NMR spectrum of 7d.

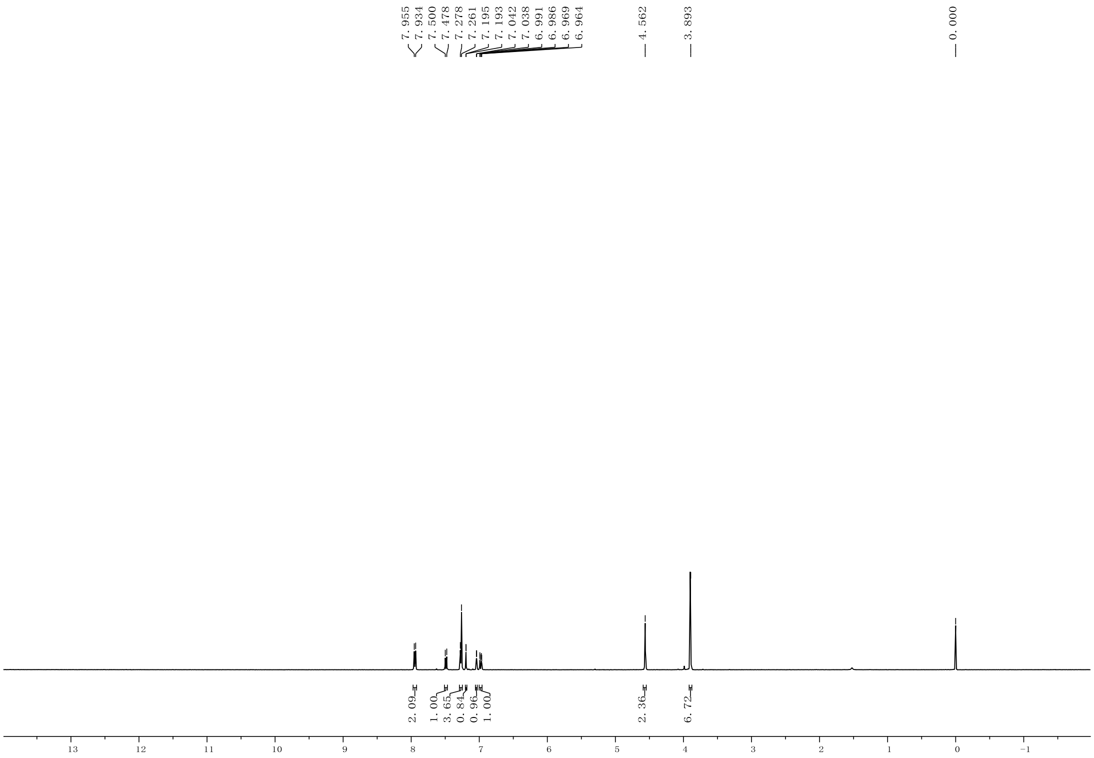

Figure S27. ^{1}H NMR spectrum of 7e.

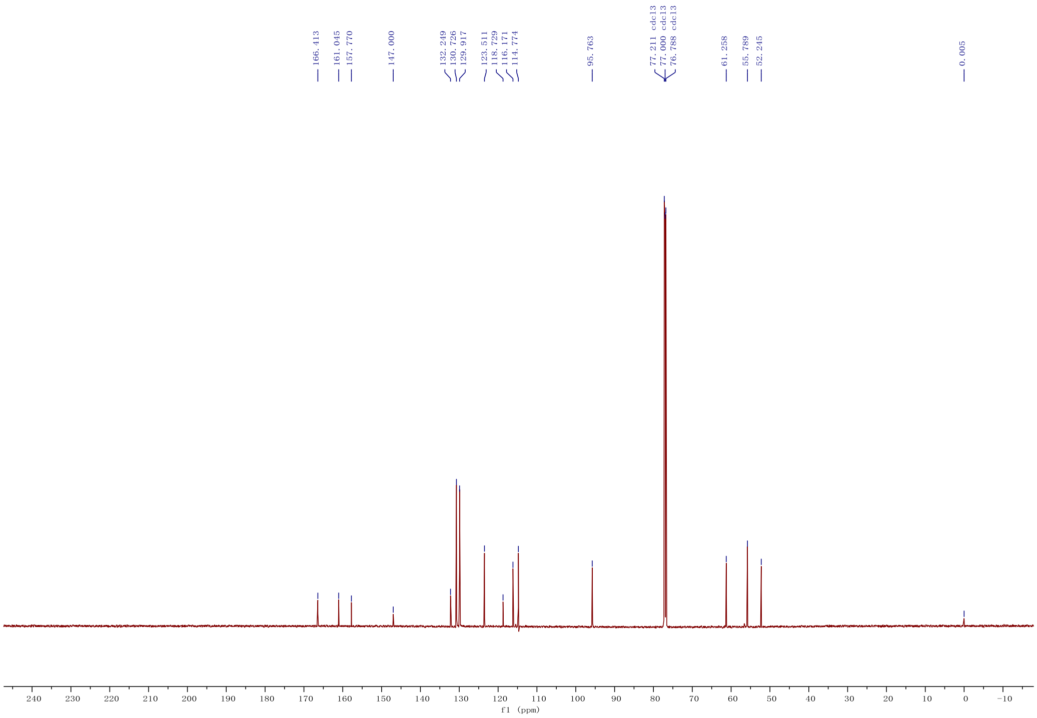

Figure S28. ^{13}C NMR spectrum of 7e.

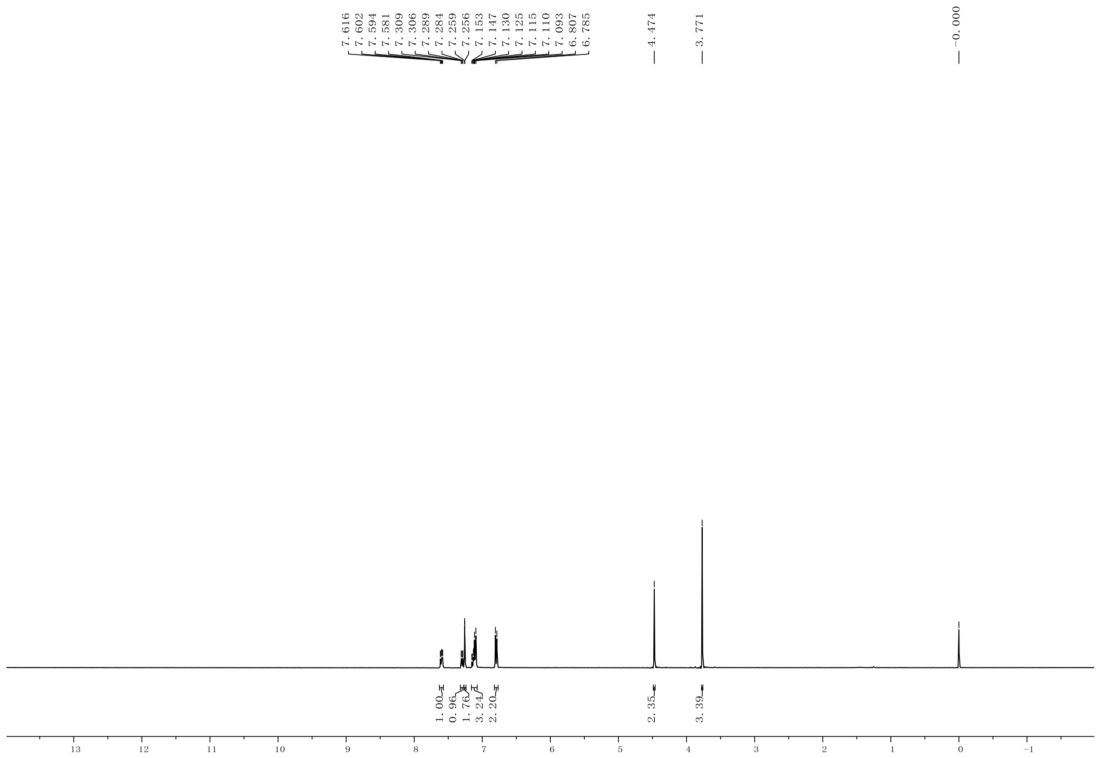

Figure S29. ^{1}H NMR spectrum of 7f.

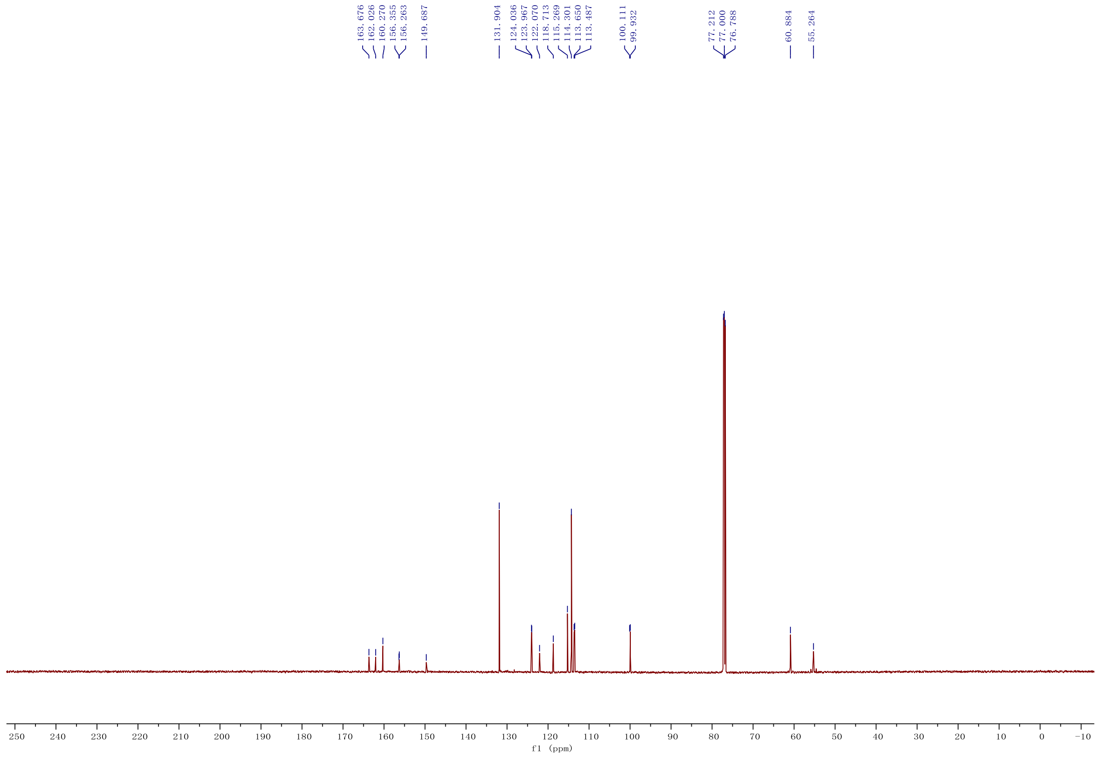

Figure S30. ^{13}C NMR spectrum of 7f.

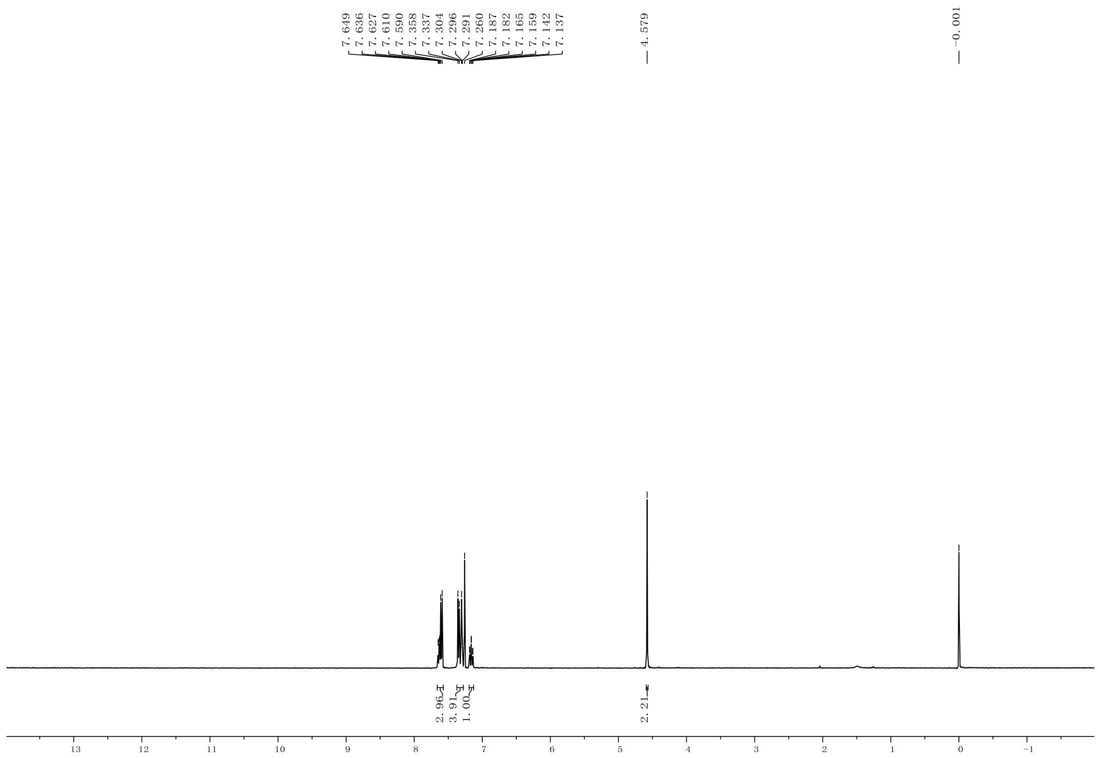

Figure S31. ^{1}H NMR spectrum of 7g.

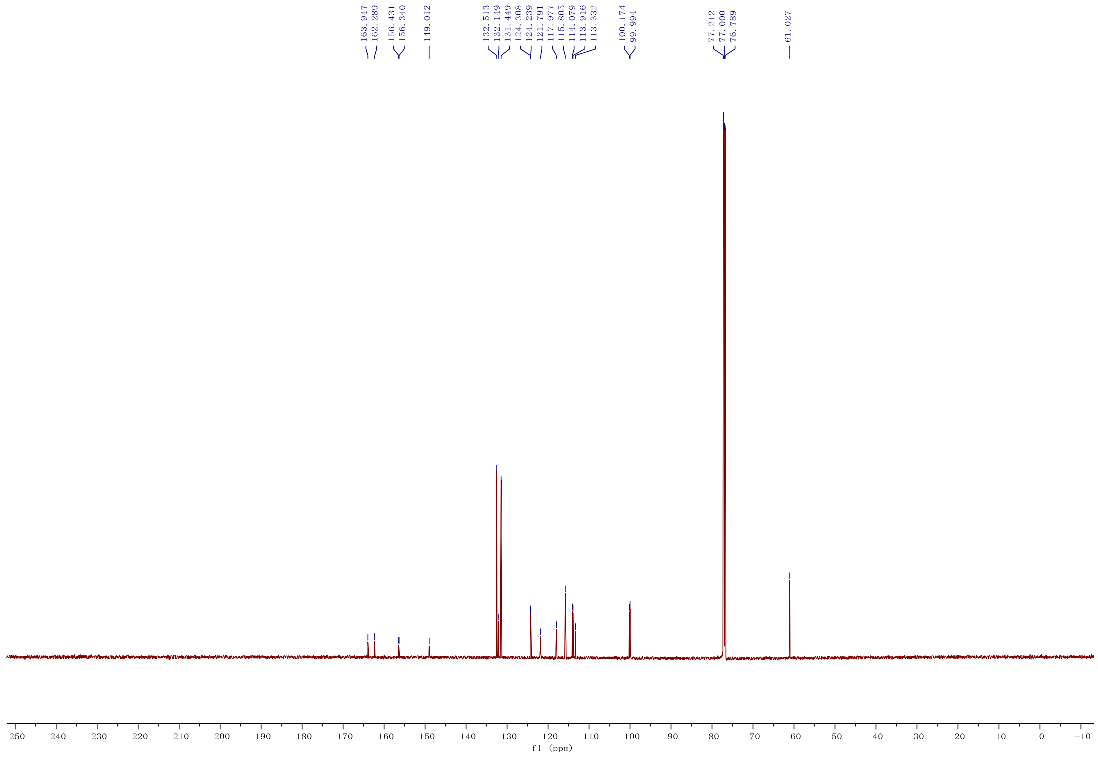

Figure S32. ^{13}C NMR spectrum of 7g.

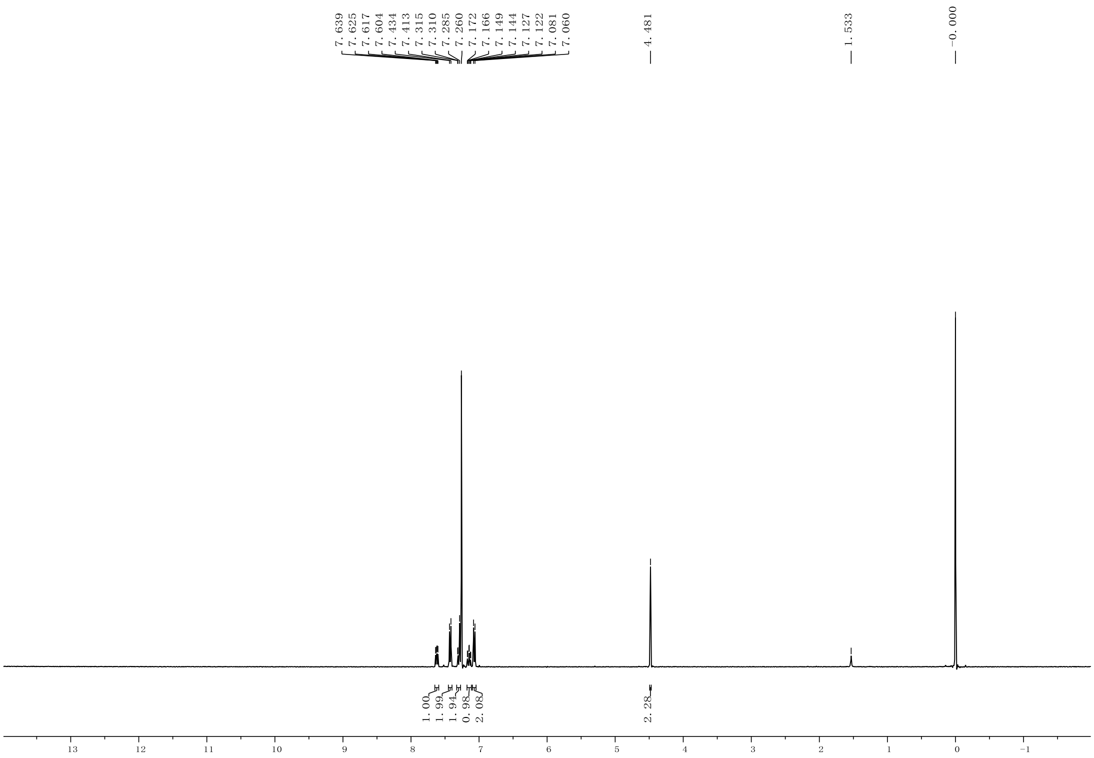

Figure S33. ^{1}H NMR spectrum of 7h.

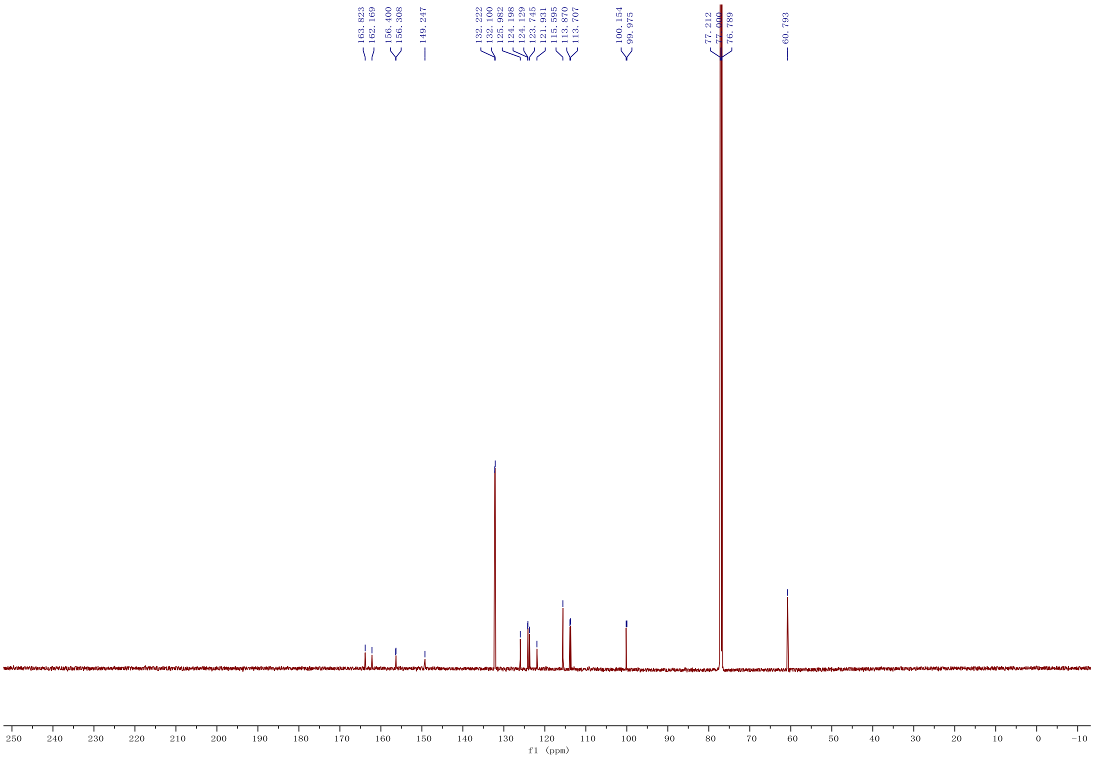

Figure S34. ^{13}C NMR spectrum of 7h.

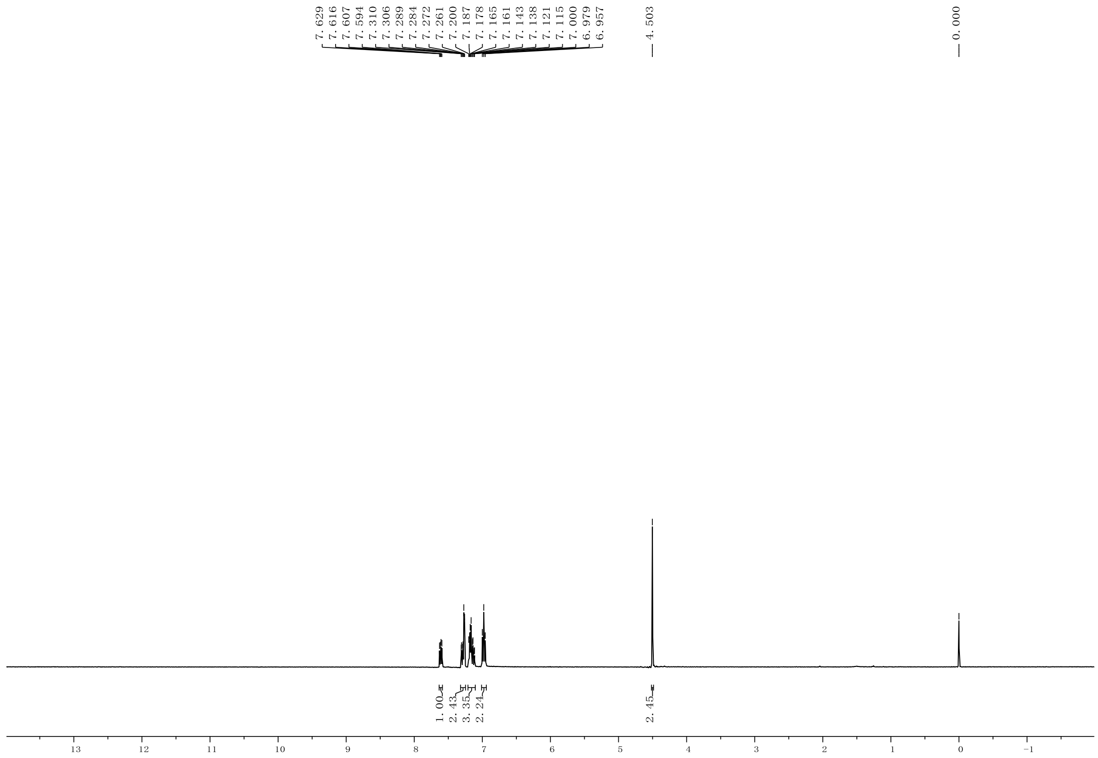

Figure S35. ^{1}H NMR spectrum of 7i.

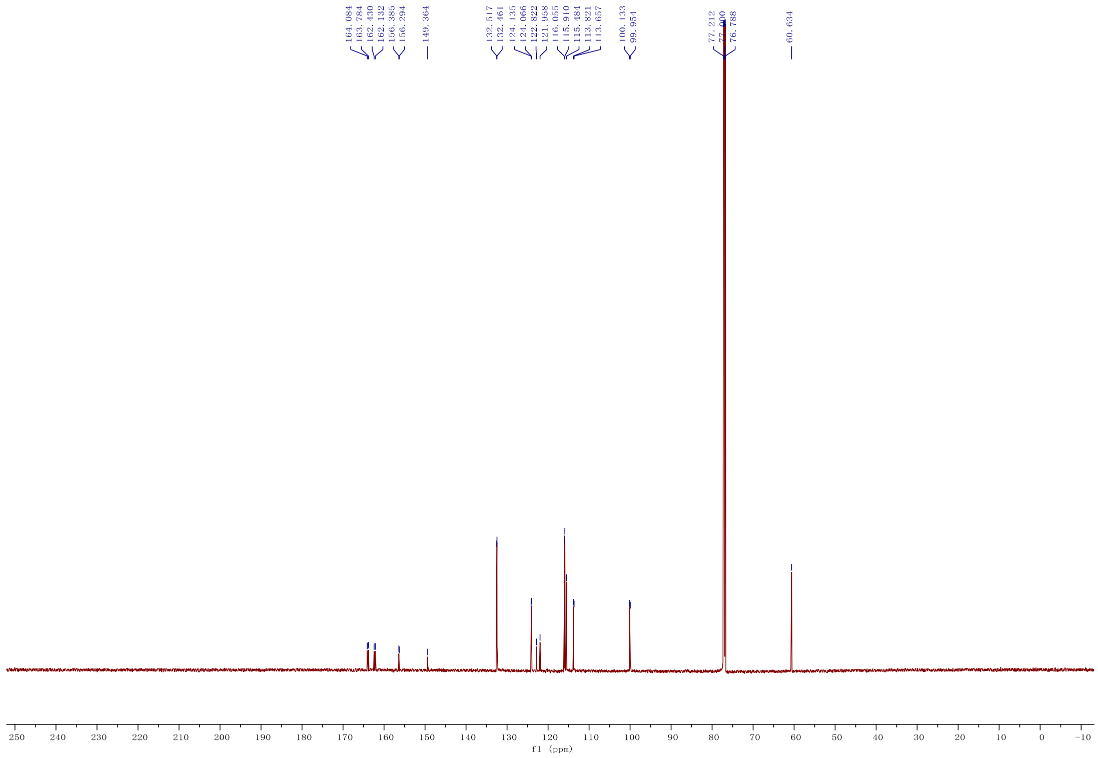

Figure S36. ^{13}C NMR spectrum of 7i.

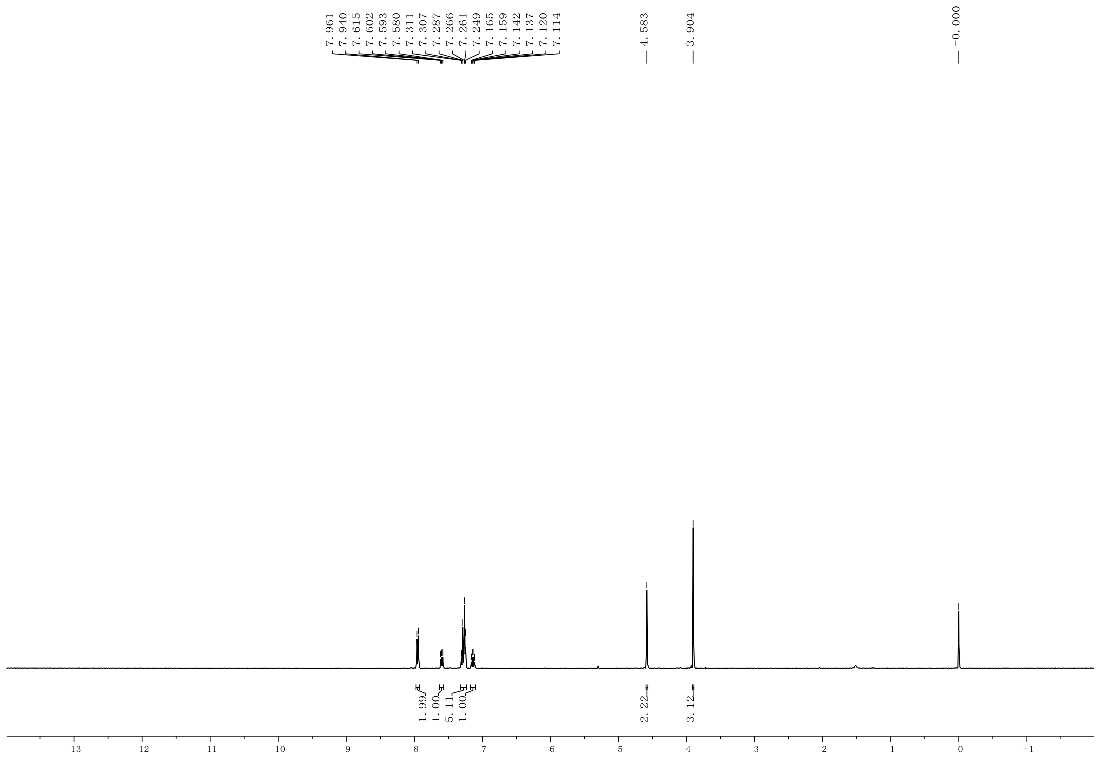

Figure S37. ^{1}H NMR spectrum of 7j.

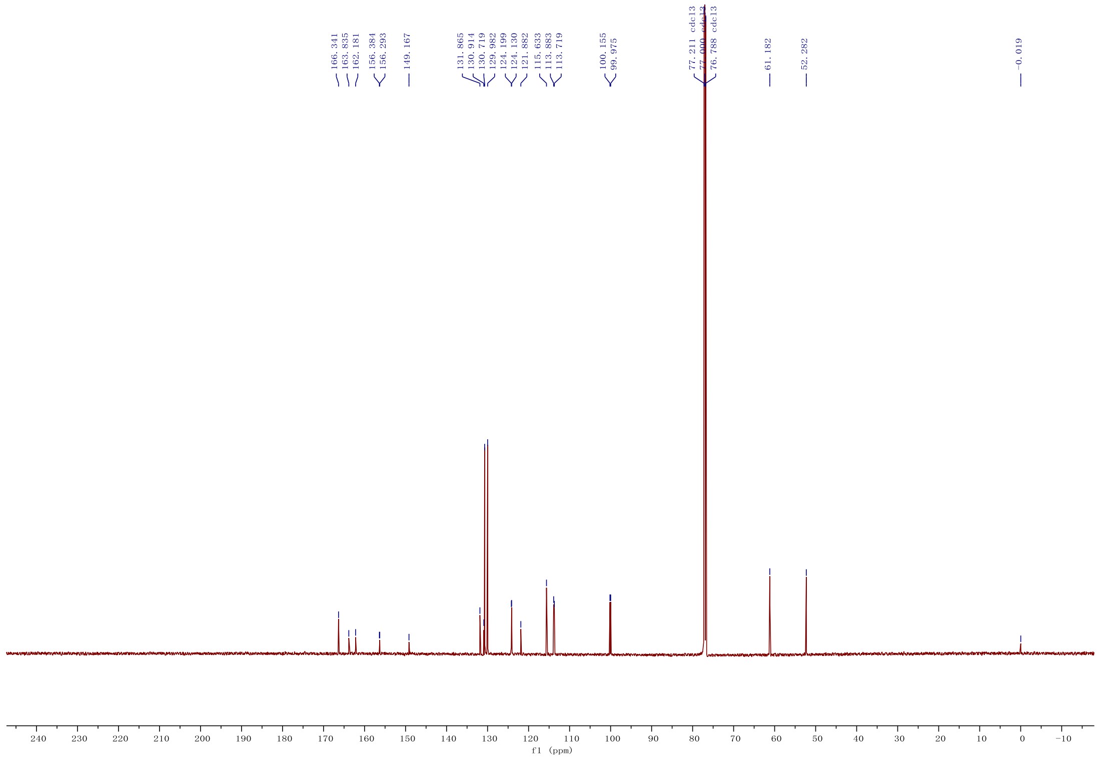

Figure S38. ^{13}C NMR spectrum of 7j.
